# Unilateral Balance, Muscular Strength, Foot Arch Status, and Physical Performance in Young Adults with and Without Low Back Pain

**DOI:** 10.3390/jcm15176648

**Published:** 2026-08-28

**Authors:** Mansour Abdullah Alshehri, Hammad Alhasan

**Affiliations:** Department of Medical Rehabilitation Sciences, Faculty of Applied Medical Sciences, Umm Al-Qura University, Makkah 24382, Saudi Arabia; hshasan@uqu.edu.sa

**Keywords:** low back pain, postural control, unilateral balance, single-leg stance, handgrip strength, foot posture, physical performance, walking, sit-to-stand

## Abstract

**Background/Objectives**: Low back pain (LBP) is a major cause of disability worldwide. In Saudi Arabia, research has predominantly focused on its prevalence and associated risk factors, with comparatively limited evidence directly comparing balance, muscular strength, foot characteristics, and physical performance between young adults with and without LBP. This study therefore compared unilateral balance and handgrip strength between young adults with and without LBP, with foot arch status, walking performance, and lower limb functional capacity examined as secondary outcomes. **Methods**: This study employed a cross-sectional observational design and enrolled 55 young participants (25 with LBP and 30 healthy controls). The primary outcomes included unilateral balance, measured using the Single-Leg Stance Test (SLST) with eyes open and eyes closed, and handgrip strength. Secondary outcomes included foot arch status, assessed using the Sit-to-Stand Navicular Drop Test (SSNDT), walking performance using the 10-Metre Walk Test (10-MWT), and lower limb functional capacity using the 30-s Chair Stand Test (30s-CST). SLST duration was analysed using generalized estimating equations, whereas multivariable linear regression models were used for the remaining outcomes. All adjusted analyses accounted for age, body mass index, sex, smoking status, athletic status, and limb dominance where appropriate. **Results**: Participants with LBP demonstrated significantly poorer unilateral balance than healthy controls after adjustment, with significant main effects of group on both the right and left SLST. Significant group-by-visual condition interactions were observed for both limbs, indicating that between-group differences were greater under eyes-closed conditions. Handgrip strength was lower in participants with LBP in the unadjusted models; however, these between-group differences were no longer significant after adjustment. No significant adjusted between-group differences were observed for SSNDT scores, walking speed, or 30s-CST performance. Among the adjustment variables, male sex, right leg dominance, and athletic status were associated with better performance, whereas increasing age and smoking were associated with poorer performance. **Conclusions**: Young adults with LBP demonstrated poorer unilateral balance than healthy controls, with between-group differences being greater under eyes-closed conditions. In contrast, no significant adjusted between-group differences were observed for handgrip strength, foot arch status, walking performance, or lower limb functional capacity. These findings identify unilateral balance, particularly under reduced visual input, as a functional domain that differed between groups in this young adult sample.

## 1. Introduction

Low back pain (LBP) represents a major global health challenge, affecting hundreds of millions of individuals and ranking as the leading contributor to years lived with disability, with considerable health and socioeconomic consequences worldwide [1,2,3]. In 2020, approximately 619 million individuals worldwide had LBP, with the number expected to reach 843 million by 2050 [3]. Although considerable progress has been made in understanding the epidemiology and clinical management of LBP, patient outcomes have improved only marginally [3,4]. This limited progress reflects the multifactorial and clinically heterogeneous nature of the condition, which is shaped by biopsychosocial factors, comorbidities, and altered pain-processing mechanisms [4]. Consequently, most cases are classified as non-specific LBP because no identifiable pathoanatomical source sufficiently explains the clinical presentation [4,5]. A better understanding of the functional and biomechanical manifestations associated with LBP may therefore improve clinical characterisation, identify clinically relevant impairments, and support more targeted rehabilitation strategies.

Saudi Arabia also experiences a considerable LBP burden, with studies documenting widespread occurrence among community-based, occupational, and educational populations [6,7,8,9]. A national systematic review and meta-analysis of 124 Saudi studies estimated pooled point, weekly, annual, and lifetime prevalence rates of 26.7%, 27.2%, 52.3%, and 64.0%, respectively, with females and healthcare workers experiencing the greatest burden [6]. Consistent findings have been reported among healthcare workers, with pooled weekly, annual, and lifetime prevalence estimates of 40.8%, 65.0%, and 81.4%, respectively [8], university students and academic staff, with an overall pooled prevalence of 57% [7], and working-age professional populations, in whom prevalence ranges from 64% to 89% [9]. In addition to its high prevalence, LBP in Saudi populations has been associated with advancing age, greater adiposity, being female, occupational workload, prolonged working hours, poor workplace ergonomics, manual handling, lifting activities, repetitive bending and twisting, and other occupational ergonomic exposures [6,7,8]. Although previous Saudi studies have substantially advanced understanding of the prevalence and risk factors of LBP, considerably less attention has been directed towards its functional and biomechanical characteristics.

The efficient and safe performance of everyday activities depends on the coordinated integration of sensorimotor control and muscular performance, which together underpin balance and physical function. Unilateral balance is particularly important because walking, stair negotiation, turning, and other functional tasks require repeated periods of single-limb support [10,11,12]. The Single-Leg Stance Test (SLST) is a valid, reliable, and widely utilised clinical assessment of unipedal balance that challenges postural stability by substantially reducing the base of support [10,11,12]. Successful performance depends on continuous integration of visual, vestibular, and somatosensory inputs [13], together with coordinated activation of the trunk and lower limb musculature to control the body’s centre of mass and maintain postural stability [14,15,16]. Assessing the balance task with and without visual input further increases the postural demands by reducing reliance on vision and increasing dependence on vestibular and somatosensory systems [10,17]. Complementing balance assessment, handgrip strength is a simple, reliable, and inexpensive surrogate measure of overall muscular strength and physical function [18,19,20,21,22,23]. Reduced handgrip strength has consistently been associated with impaired physical performance, disability, sarcopenia, cardiovascular disease, hospitalisation, and all-cause mortality, supporting its value as an integrated indicator of musculoskeletal health and functional capacity [18,19,22,23]. Collectively, the SLST and handgrip strength provide complementary assessments of postural control and global muscular performance and were therefore selected as the primary outcome measures in the present study.

Beyond postural control and overall muscular strength, foot biomechanics and physical performance may also contribute to the functional presentation of LBP. Foot posture influences lower limb alignment, plantar loading, and postural stability during weight-bearing activities [24,25,26]. The Sit-to-Stand Navicular Drop Test (SSNDT) provides a simple, reliable clinical measure of medial longitudinal arch (MLA) function by quantifying dynamic changes in navicular height during weight bearing [27,28]. Excessive foot pronation, reflected by greater navicular displacement, has been associated with altered lower limb biomechanics and several musculoskeletal disorders [29,30], including LBP [31,32,33]. Physical performance represents another important dimension of function, with habitual walking speed [34] and lower limb functional capacity [35] widely regarded as reliable measures of mobility, functional status, and overall health. These domains are commonly assessed using the 10-Metre Walk Test (10-MWT) [36] and the 30-Second Chair Stand Test (30s-CST) [35,37], respectively. Accordingly, the SSNDT, 10-MWT, and 30s-CST were included as secondary outcome measures in the present study.

Despite the substantial epidemiological evidence describing the burden of LBP in Saudi Arabia, important gaps remain in understanding its functional manifestations. Existing Saudi studies have predominantly focused on prevalence, associated factors, clinical practice, or healthcare professionals’ knowledge of LBP, whereas evaluation of clinical and functional outcomes in individuals with LBP has received little attention. Although previous Saudi evidence has examined postural control and hip muscle strength in individuals with LBP, evidence regarding foot posture remains limited [38], and the concurrent evaluation of multiple functional domains within the same cohort has received less attention. The present study therefore extends existing evidence through a multidimensional assessment encompassing unipedal balance with and without visual input, handgrip strength, foot arch status, walking performance, and lower limb functional capacity. Interpretation of some of the existing international evidence is further limited by inconsistent adjustment for key potential confounders, including age, sex, body mass index (BMI), smoking status, athletic status, and dominant limb, despite their recognised influence on postural control, muscular strength, foot biomechanics, and physical performance. In addition, assessment under reduced visual input (eyes closed) may impose greater postural demands than assessment with visual input available (eyes open), potentially revealing balance impairments that are less apparent under eyes-open conditions [39]. The limited evidence in these areas is particularly relevant in Saudi Arabia, where LBP represents a considerable public health burden and evidence regarding its associated functional characteristics remains limited. A better understanding of differences in unilateral balance, muscular strength, walking performance, foot arch status, and lower limb functional capacity between individuals with and without LBP may improve clinical assessment, facilitate the identification of functional deficits, and inform more individualized approaches to rehabilitation and prevention.

Accordingly, this study investigated whether individuals with LBP differ from healthy controls across a range of functional and biomechanical characteristics. The primary aims were to compare unilateral balance performance with and without visual input and handgrip strength between individuals with and without LBP. The secondary aims were to compare foot arch status, walking performance, and lower limb functional capacity between individuals with and without LBP. To minimise the influence of potential confounding factors, all comparisons were adjusted, as appropriate, for age, sex, BMI, smoking status, athletic status, and limb dominance.

## 2. Materials and Methods

### 2.1. Design, Setting, and Ethical Considerations

A cross-sectional observational design was implemented within the Biomechanics Laboratory, Department of Medical Rehabilitation Sciences, Faculty of Applied Medical Sciences, Umm Al-Qura University, Makkah, Saudi Arabia. Participant recruitment and data collection took place between February and April 2026. Recruitment relied on convenience sampling, with potential participants approached through online advertisements, posted notices, and outreach across the university community. Given the university-based recruitment setting, the recruited sample was predominantly composed of young adults, with all participants except three aged 18–25 years. Participants who expressed interest in participation underwent eligibility screening before enrolment.

Ethical clearance was granted by the Institutional Review Board of Umm Al-Qura University (Approval No. HAPO-02-K-012-2025-12-3052). All study procedures adhered to the principles of the Declaration of Helsinki, and each participant provided written informed consent prior to enrolment. All data were anonymized before analysis. Participation was entirely voluntary, and participants could discontinue their involvement at any stage without penalty. Reporting of the study followed the Strengthening the Reporting of Observational Studies in Epidemiology (STROBE) guidelines.

### 2.2. Eligibility Criteria

Participants were considered eligible if they were aged ≥18 years and capable of independent standing and ambulation without physical assistance. Participants were subsequently classified into the LBP or control group. LBP was defined as pain localised between the 12th thoracic vertebra and the gluteal fold that persisted for at least 24 h during the preceding week. The Numeric Pain Rating Scale (NPRS) was used to quantify pain severity. Participants in the LBP group were required to report an NPRS score of ≥1 at the time of assessment. Participants were classified according to the presence of LBP during the specified assessment period rather than according to symptom duration or clinical stage. Control participants were required to be healthy and free from LBP and related disability.

In both groups, eligibility required the absence of health conditions, other than LBP, that might interfere with the outcomes being evaluated. Participants were excluded if they had experienced lower limb trauma, including injury or fracture, or had undergone surgery within the previous six months; had a vestibular disorder affecting balance or spatial orientation; had a severe musculoskeletal deformity involving foot posture, lower limb alignment, or mobility; or had neurological, cardiovascular, respiratory, or other serious medical conditions that could compromise physical performance or interfere with study assessments. Pregnancy and the presence of foot wounds, ulcers, or any other condition preventing barefoot assessment were additional grounds for exclusion.

### 2.3. Assessment Standardization Procedures

The complete assessment protocol was performed in the laboratory within one session of approximately 60 min. Prior to participant enrolment, physiotherapists involved in data collection received training in the study protocol and assessment procedures. Assessment responsibilities were predefined and remained unchanged throughout the study. One physiotherapist collected demographic information and LBP-related data. Another physiotherapist carried out all handgrip strength and SSNDT measurements. Balance and physical performance assessments, including the SLST, 10-MWT, and 30s-CST, were conducted by two physiotherapists, with one overseeing participant instructions and safety and the other recording assessment data. Assessors were not blinded to participants’ LBP status; however, all assessments were conducted according to predefined standardised procedures and criteria to minimise potential measurement bias. Participants performed all outcome assessments without footwear to limit its potential effects on foot posture [27], as well as on balance and physical performance [10,40].

### 2.4. Outcome Measures

Prior to outcome assessment, participants underwent demographic and anthropometric evaluation. Information recorded involved age, sex, nationality, marital status, residential location, educational attainment, athletic participation status (i.e., routine engagement in sports or organized exercise programs), dominant hand, and dominant lower limb. Anthropometric profiling consisted of standing height and body weight, with height recorded using a fixed wall stadiometer and weight obtained from a calibrated digital scale. BMI was then calculated as body weight in kilograms divided by the square of height in metres (kg/m^2^).

#### 2.4.1. Unilateral Balance Performance

Unilateral balance was evaluated using the SLST. Participants completed the test with eyes open and eyes closed to assess balance under different visual conditions. This approach was used because vision contributes substantially to postural control, and unilateral balance performance varies when visual information is available or removed [10,41]. Testing procedures were primarily based on a previously published normative SLST protocol for healthy adults [10]. Evidence supporting the reliability of the SLST has been reported in several populations. In healthy adults, inter-rater reliability was excellent for testing with eyes open (intraclass correlation coefficient [ICC] = 0.95) and eyes closed (ICC = 0.83) [10]. Similarly, high inter-rater agreement has been observed in uninjured athletes (κ = 0.90) [40] and individuals with LBP (right foot κ = 1.00; left foot κ = 0.88) [42]. Among individuals with neurological conditions, test–retest reliability was good to excellent for both the right (ICC = 0.82) and left (ICC = 0.83) limbs [43].

**Figure 1:**
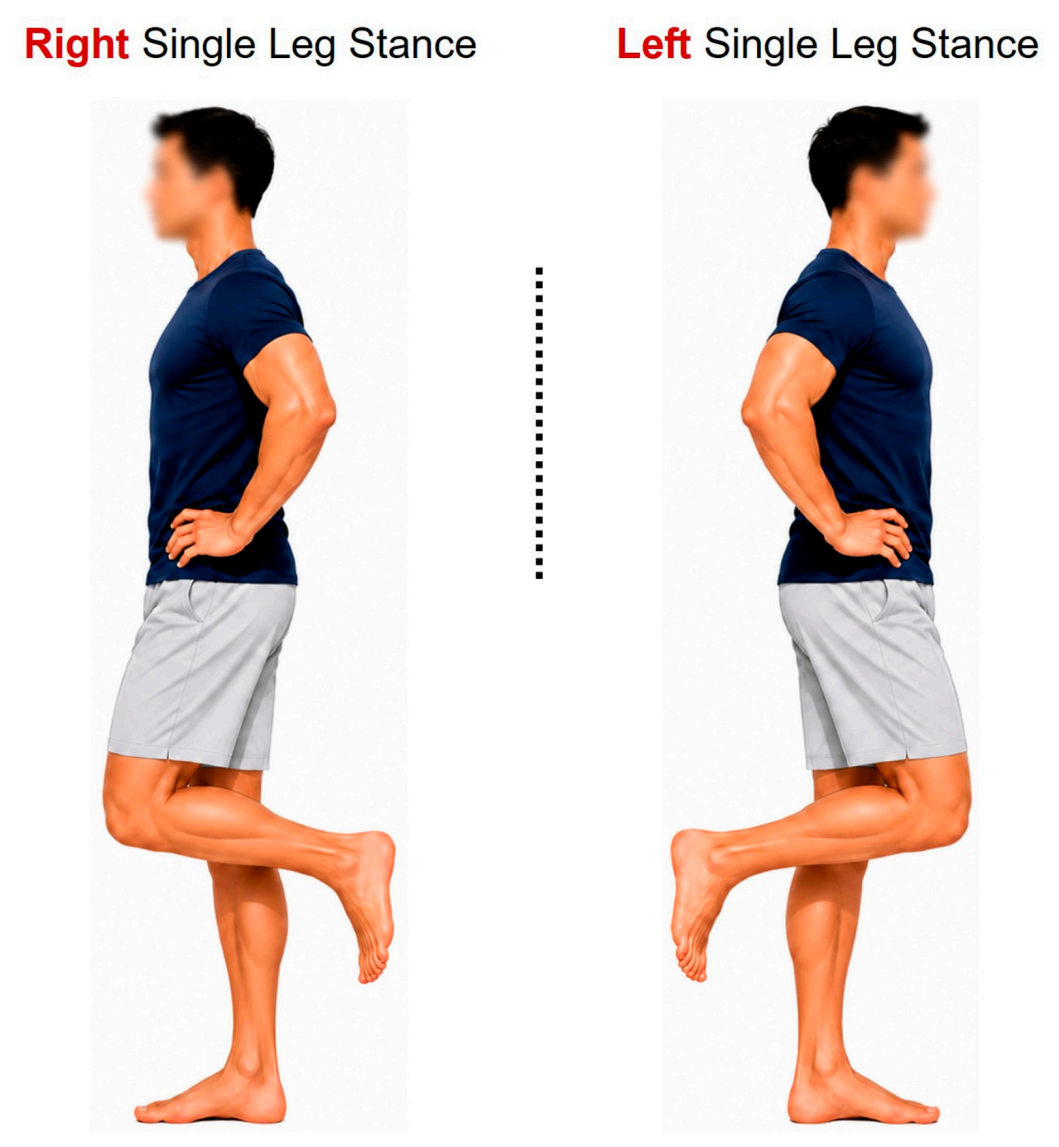
SLST positions during right and left unilateral stance assessments. Participants completed the test barefoot, with their hands positioned on the hips and the non-supporting limb held at approximately 90° of knee flexion. Note: SLST = single-leg stance test.

Participants performed the test barefoot while standing on a single leg, with their hands resting on their hips. The stance limb was maintained in full knee extension, whereas the opposite limb was flexed to approximately 90° at the knee (Figure 1). Both limbs were evaluated separately with vision available and with the eyes closed. For testing with eyes open, participants maintained their focus on a fixed point at eye height located approximately 2 m in front of them. For testing with eyes closed, participants first directed their gaze toward the same fixed point and then closed their eyes just before the trial began. Throughout all trials, participants were asked to hold the required position with minimal body movement. The instruction given was to “maintain your balance as still as possible.”

For safety purposes, a chair was placed immediately in front of each participant, and a physiotherapist remained in close proximity during testing. Before the recorded assessments, participants performed a separate familiarization attempt for each of the four testing conditions: right limb with eyes open, right limb with eyes closed, left limb with eyes open, and left limb with eyes closed. Following each familiarization attempt, participants completed three recorded trials of the corresponding condition, with each trial limited to a maximum of 30 s. A 30-s recovery interval separated each trial to reduce any effect of fatigue on performance.

SLST performance was quantified as the time, in seconds, for which the participant maintained the unipedal stance before balance was lost or a compensatory movement occurred. The trial ended when the participant shifted or rotated the supporting foot, touched the floor or supporting limb with the raised foot, moved either hand away from the hips, opened the eyes during the eyes-closed testing, or reached the 30-s time limit [10,44]. For each trial, the elapsed time from trial initiation until termination was recorded, irrespective of whether termination resulted from loss of balance, a compensatory movement, failure to maintain the prescribed testing position, or completion of the maximum test duration. For each limb and visual condition, the mean of the three recorded SLST trial durations was calculated and used in the statistical analyses. Stance duration has been widely used as a measure of unilateral balance performance in studies involving healthy young adults [10,12,45], older adults [10,44,45,46], and individuals with clinical conditions [43,47].

#### 2.4.2. Handgrip Strength

**Figure 2:**
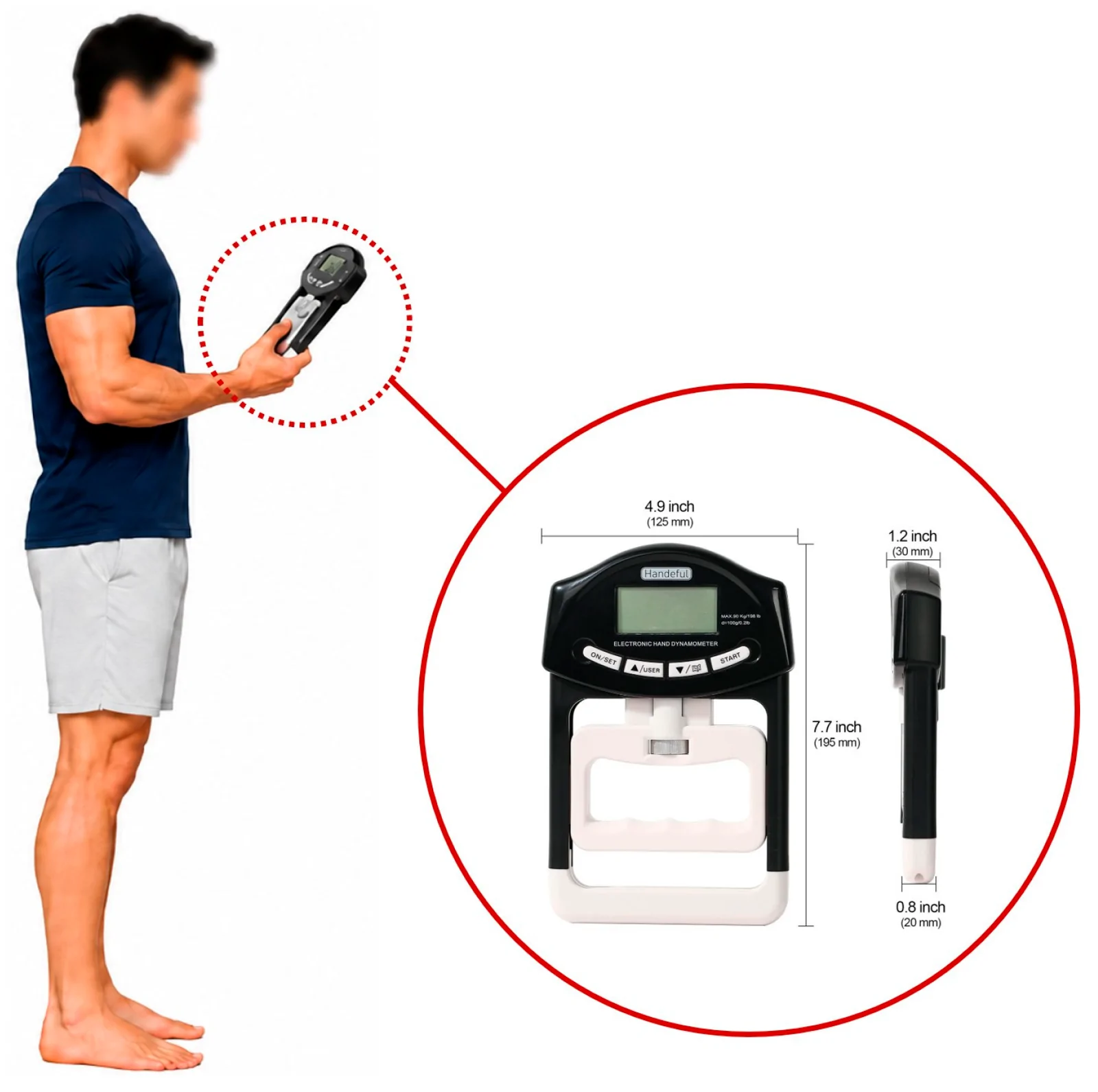
Participant posture and upper limb position during handgrip strength measurement using a digital hand dynamometer.

Overall muscular strength was estimated indirectly from handgrip force measured with a digital hand dynamometer (HFEH11BK; Handeful, China; Figure 2). While the Jamar dynamometer is commonly considered the benchmark instrument for measuring handgrip strength [48,49,50], evidence supports the measurement properties of digital dynamometers, with excellent repeatability (ICC = 0.94–0.95) [51] and good agreement with Jamar-derived values (ICC = 0.82–0.83) [52].

Handgrip strength testing (Figure 2) was performed with participants while standing upright. The tested limb was positioned alongside the trunk, with the elbow flexed to 90°, the forearm in supination, and the wrist maintained in a neutral position [49]. Before formal testing, participants completed one practice trial with each hand and received standardised verbal instructions. Participants were asked to refrain from using compensatory trunk movements and any additional upper limb movements during maximal grip exertion. Measurements were obtained separately for the right and left hands. Participants were instructed to exert maximal grip force and maintain the contraction for 5 s. A 60-s recovery interval separated the assessments to minimise the potential influence of fatigue. For each hand, grip strength was quantified as the maximum force generated during testing, expressed in kilograms.

#### 2.4.3. Foot Arch Status

The SSNDT was used to assess foot arch status [27], a modified form of the navicular drop test originally described by Brody in 1982 [53]. The SSNDT measures the change in navicular tuberosity position between non-weight-bearing and weight-bearing conditions, thereby reflecting functional changes in MLA height. As such, it is commonly used as a clinical measure of foot pronation [27]. Previous studies in healthy adults have reported moderate reliability for the SSNDT, with estimates of 0.68–0.70 for intra-rater reliability and 0.72 for inter-rater reliability based on ICCs [27]. In physically active healthy adults, the SSNDT has also demonstrated good within-day intra-rater reliability (ICC = 0.82) [54].

**Figure 3:**
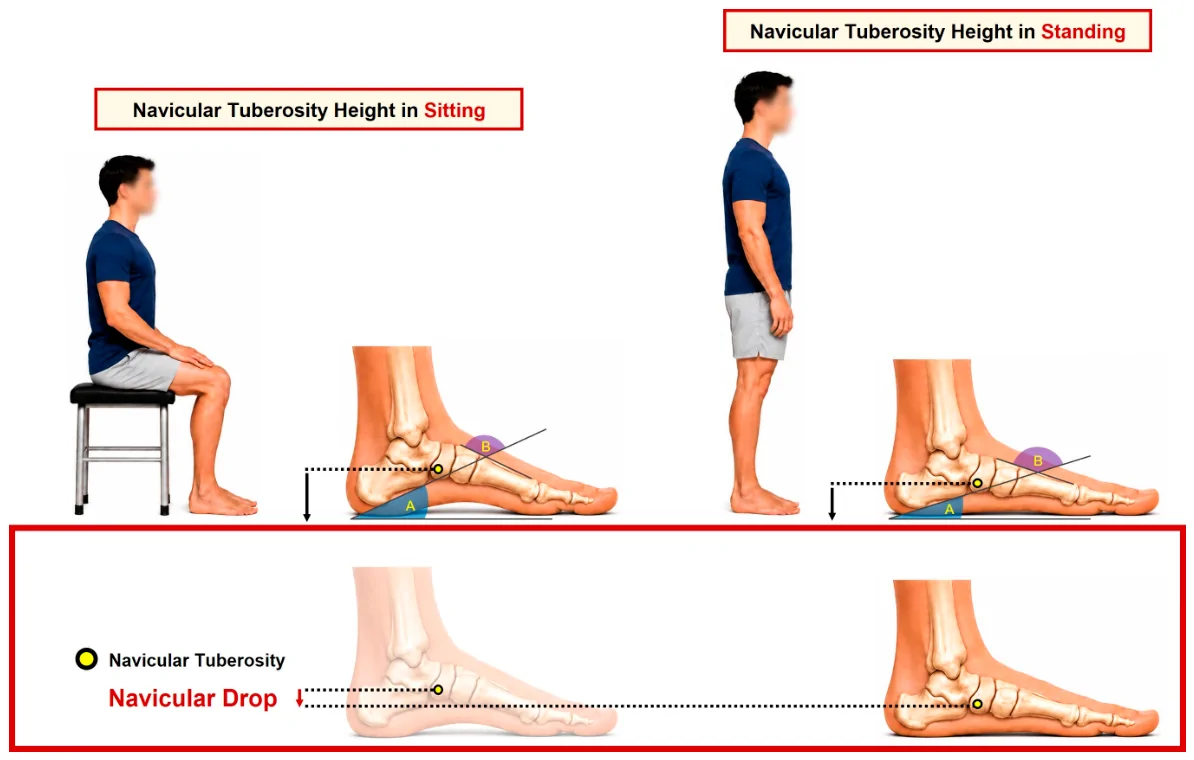
SSNDT procedure with a schematic representation of foot alignment in the sagittal plane. The SSNDT value represents the change in navicular tuberosity height between seated and relaxed standing measurements. Larger values reflect greater lowering of the MLA during weight bearing and a more pronated foot posture. The schematic depicts the calcaneal inclination angle (A) and the calcaneal-first metatarsal angle (B). A smaller calcaneal inclination angle together with a larger calcaneal-first metatarsal angle reflects a lower MLA and increased foot pronation. Note: SSNDT = Sit-to-Stand Navicular Drop Test; MLA = medial longitudinal arch.

The assessment began with participants sitting on a chair, with both the hips and knees positioned at approximately 90° of flexion and the ankles held in neutral. The navicular tuberosity was palpated to locate its most prominent point, which was subsequently marked. Using a ruler positioned perpendicular to the floor, the vertical distance between this landmark and the supporting surface was recorded as the navicular height. Following the seated assessment, participants assumed a relaxed bipedal standing position with body weight distributed evenly between both lower limbs. Navicular height was then re-measured using the same procedure described for the seated position (Figure 3). Each foot was assessed independently. The SSNDT value, reported in millimetres, was determined by subtracting the standing navicular height from the seated measurement. Higher SSNDT values suggested a greater reduction in MLA height during weight bearing and were interpreted as reflecting a more pronated foot posture [27,53].

#### 2.4.4. Lower Limb Functional Capacity

The 30s-CST was used to evaluate functional capacity of the lower limbs. Evidence from different populations, including community-dwelling older adults and people with knee osteoarthritis, indicates good-to-excellent reproducibility of this test, with test–retest ICCs between 0.84 and 0.94 [35,55].

Participants were seated on a standardised armless chair with both feet supported on the floor and the hips and knees flexed to approximately 90°. When required, the chair height was modified to maintain comparable lower limb positioning among participants. Before the recorded assessment, participants were given uniform instructions and performed a single familiarization attempt to become accustomed to the test protocol.

**Figure 4:**
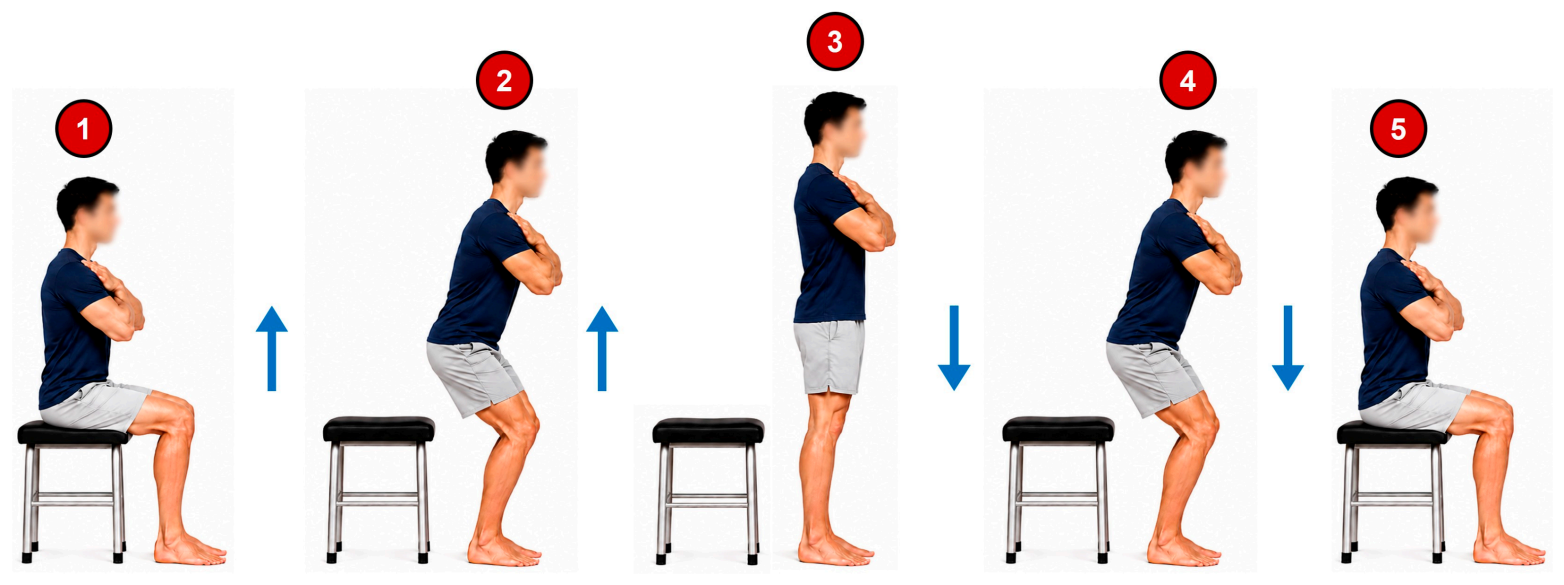
Sequential testing positions of the 30s-CST, including (1) the seated position, (2) the transition from sitting to standing, (3) the full standing position, (4) the transition from standing to sitting, and (5) the return to the seated position. Note: 30s-CST = 30-Second Chair Stand Test.

Participants kept their arms folded across the chest while completing the greatest possible number of sit-to-stand repetitions during the 30-s test period. Each repetition consisted of rising from the seated position to full standing and subsequently returning to the seated position (Figure 4). Only fully completed sit-to-stand-to-sit cycles were recorded as successful repetitions. Performance was quantified by the number of repetitions successfully completed within 30 s. When the 30-s limit was reached during an ongoing repetition, the repetition was included if at least half of the movement had already been performed [37].

#### 2.4.5. Walking Performance

The 10-MWT was used to evaluate habitual walking performance, a widely used measure of locomotor function and usual walking speed [38]. The 10-MWT has demonstrated high test–retest reliability in both healthy young and older adults, with reported ICCs ranging from 0.97 to 0.98 [36,56].

**Figure 5:**
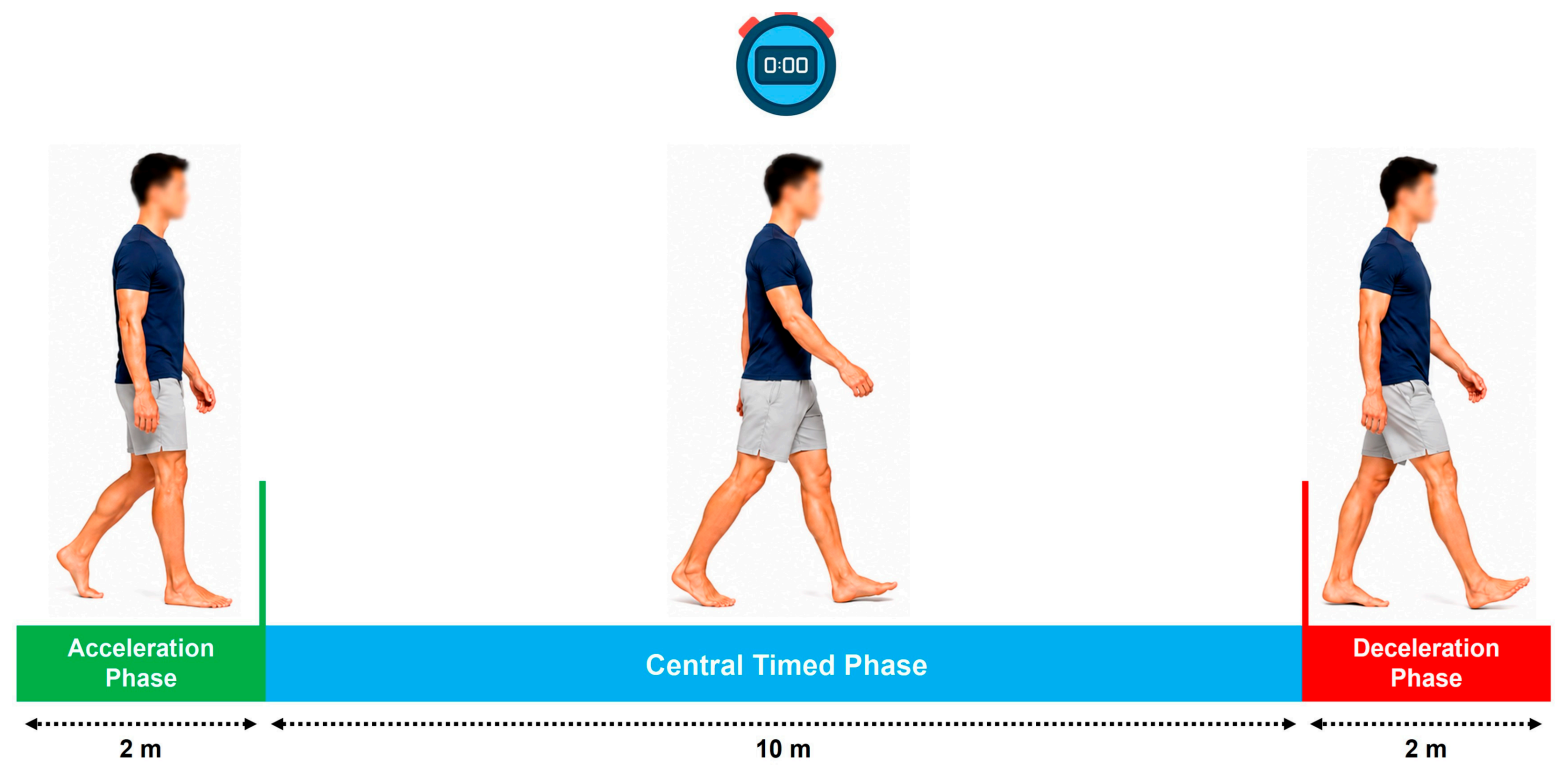
Schematic representation of the 10-MWT assessment protocol. Participants walked along a 14-m walkway consisting of an initial 2-m section for acceleration, a middle 10-m section for timed walking, and a final 2-m section for deceleration. Note: 10-MWT = 10-Metre Walk Test.

Participants traversed a 14-m walkway at their normal, self-selected walking pace. The 14-m walkway included an initial 2-m acceleration distance, a central 10-m distance over which walking time was recorded, and a final 2-m deceleration distance (Figure 5). A stopwatch was used to record the time required to traverse the central 10-m distance. Timing commenced when the leading foot crossed the 2-m marker and ceased upon crossing the 12-m marker, which represented the start and end points of the timed distance, respectively [36]. Walking speed was computed as the 10-m timed distance divided by the time taken to cover that distance, with values reported in metres per second (m/s).

### 2.5. Power Analysis

A post-hoc power calculation was performed in G*Power (version 3.1.9.7; Heinrich Heine University Düsseldorf, Düsseldorf, Germany) to determine the statistical power achieved with the available sample. As the primary analyses examined differences between participants with and without LBP in continuously measured outcomes while accounting for relevant covariates and fixed factors, statistical power was estimated using an F-test based on the R^2^ increase approach. The analysis specified group status (LBP versus control) as the predictor of interest, with age, BMI, sex, smoking status, athletic status, and dominant limb entered as six adjustment variables, resulting in seven predictors overall. With a moderate effect size (f^2^ = 0.15), α = 0.05 for a two-sided test, and an analytical sample of 55 participants, the estimated power reached 80%. Accordingly, the final sample was deemed adequate for identifying moderate group differences in the primary adjusted multivariable analyses.

### 2.6. Data Analysis

#### 2.6.1. Descriptive Analyses

Demographic, anthropometric, lifestyle, and limb-dominance characteristics were described for the full cohort and separately for participants with and without LBP. Normally distributed continuous variables were reported as means with standard deviations (SD), whereas non-normally distributed variables were reported as medians with interquartile ranges (IQR). Categorical variables were reported as frequencies and percentages. Comparisons between participants with and without LBP were examined with independent two-sample t-tests when continuous data followed a normal distribution and Wilcoxon rank-sum tests when the distribution was non-normal, while categorical data were compared using Fisher’s exact tests.

#### 2.6.2. Confounding Variables

Potential confounding variables were selected based on previous evidence indicating their associations with the study outcome variables. Age and BMI were included as continuous covariates because both have been associated with foot posture, balance, muscular strength, and physical performance outcomes [48,57,58]. Sex was included as a categorical factor owing to reported sex-related differences in foot structure, muscular strength, and physical performance [48,57]. Athletic status was included because regular participation in sports and physical training may influence foot characteristics, neuromuscular function, and physical performance [48,54,59]. Smoking status was included as a categorical factor because smoking has been associated with adverse musculoskeletal health outcomes, including reduced muscle strength, impaired physical function, and altered balance performance [60]. Lower limb dominance was adjusted for in models involving foot arch status and balance outcomes because inter-limb differences may affect unilateral loading, postural control, and movement performance [61]. Hand dominance was included only in handgrip strength models because dominant and non-dominant hands differ in grip strength performance [49,62].

#### 2.6.3. Unilateral Balance Performance

SLST duration was analysed using generalized estimating equations (GEEs). GEEs were selected to account for the correlation between repeated SLST measurements obtained during the eyes-open and eyes-closed conditions. Separate models were performed for each limb. Separate limb-specific models were used because right- and left-limb performance was considered as distinct outcomes that may reflect side-specific biomechanical and sensorimotor characteristics. This approach also avoided the additional complexity of higher-order interactions involving group, visual condition, and limb within the relatively modest sample. SLST duration was specified as the dependent variable. Participant group (LBP versus control), visual condition (eyes open versus eyes closed), and the group-by-visual condition interaction were included as the primary model terms. Sex, smoking status, athletic status, and dominant leg were included as categorical factors, while age and BMI were included as continuous covariates.

Models were fitted using a Gaussian family with an identity link function, an exchangeable working correlation structure, and robust standard errors. Adjusted marginal means and corresponding 95% confidence intervals (CIs) were estimated for participant group, visual condition, and group-specific comparisons across visual conditions. These results were presented in marginal means plots. Significant interaction effects were followed by pairwise comparisons with Bonferroni correction. GEE coefficients and 95% CIs were presented for all model terms in the coefficient plots, including categorical factors and continuous covariates.

#### 2.6.4. Handgrip Strength and Foot Arch Status

Handgrip strength and SSNDT scores were analysed using multivariable linear regression models with robust standard errors. Separate models were performed for right and left handgrip strength and for right and left SSNDT scores. For all models, participant group (LBP versus control) was included as the primary model term. Sex, smoking status, and athletic status were included as categorical factors, whereas age and BMI were included as continuous covariates. Models evaluating handgrip strength were additionally adjusted for dominant hand, whereas models evaluating SSNDT scores were additionally adjusted for dominant leg. Both unadjusted and adjusted models were performed. Adjusted marginal means and corresponding 95% CIs were estimated from the adjusted models. These results were presented in marginal means plots. Regression coefficients and 95% CIs were also presented for all model terms in the coefficient plots, including categorical factors and continuous covariates.

#### 2.6.5. Lower Limb Functional Capacity and Walking Performance

30s-CST performance and 10-MWT walking speed were analysed using multivariable linear regression models with robust standard errors. For each outcome, participant group (LBP versus control) was included as the primary model term. Sex, smoking status, and athletic status were included as categorical factors, whereas age and BMI were included as continuous covariates. Both unadjusted and adjusted models were performed. Adjusted marginal means and corresponding 95% CIs were estimated from the adjusted models. These results were presented in marginal means plots. Regression coefficients and 95% CIs were also presented for all model terms in the coefficient plots, including categorical factors and continuous covariates.

#### 2.6.6. Statistical Significance

A *p*-value below 0.05 was considered statistically significant. Analyses were conducted in Stata/MP 17 (StataCorp LLC, College Station, TX, USA). Stata was used to produce statistical graphs and visual displays, while Microsoft PowerPoint was used to prepare the study flow diagram. Illustrative figures of the exposure and outcome assessment procedures were initially generated using an artificial intelligence-assisted image generation platform and subsequently refined, edited, and finalised using additional software. Each figure underwent review and received approval from all members of the research team.

## 3. Results

### 3.1. Participant Selection

**Figure 6:**
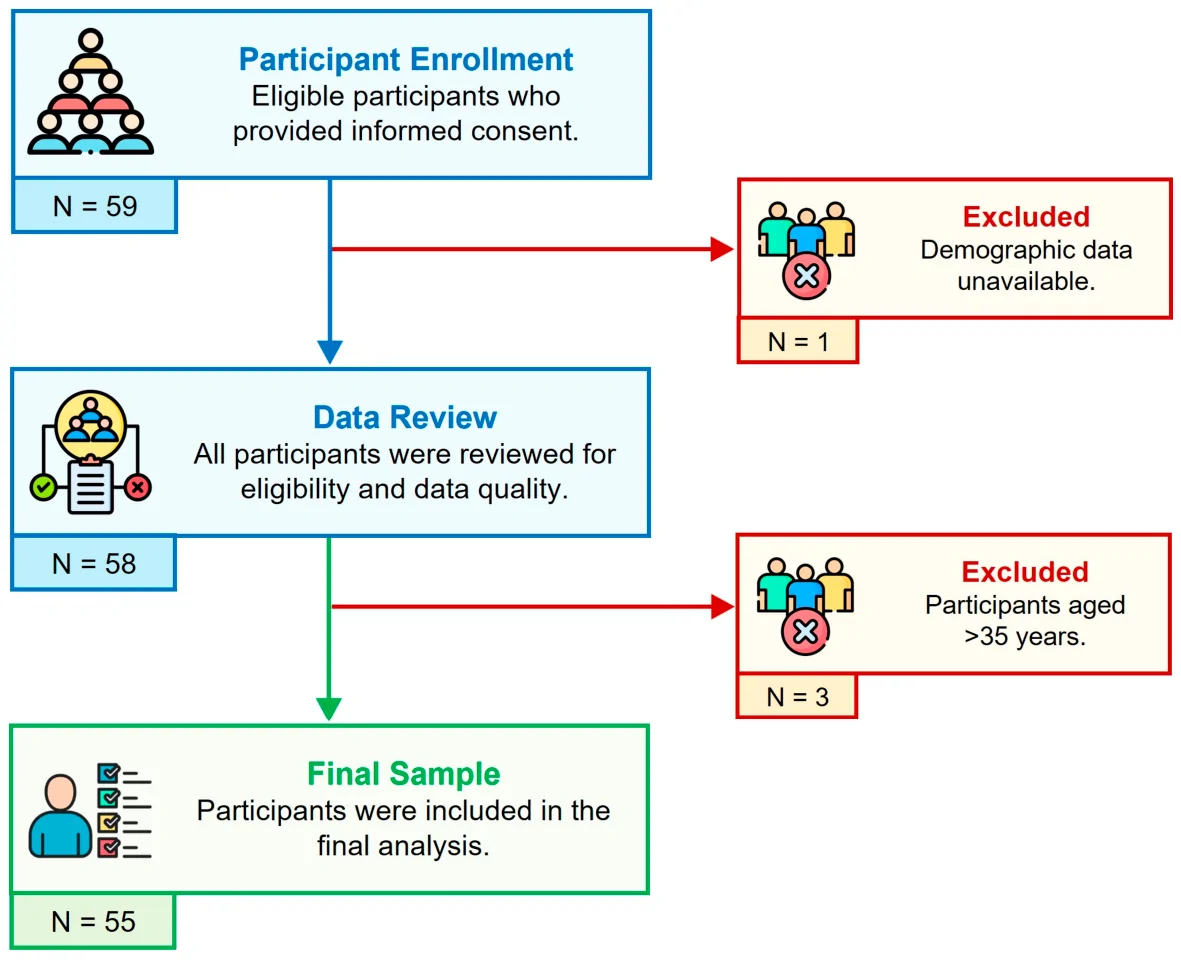
Flow diagram of study participants.

The study initially enrolled 59 participants after written informed consent had been obtained. Following data verification, one participant was excluded because demographic data were unavailable. The remaining 58 participants were assessed for eligibility and data quality. Three participants (one with LBP and two without LBP) were subsequently excluded because they were older than 35 years. The exclusion was intended to reduce possible confounding related to age and preserve a comparatively homogeneous cohort of young adults. No participants met any other predefined exclusion criteria. Consequently, 55 participants were included in the final analysis (Figure 6).

### 3.2. Participant Characteristics

There were no statistically significant between-group differences in age, height, weight, BMI, smoking status, athletic status, dominant leg, or dominant hand (all *p* > 0.05). However, sex distribution differed significantly between the two groups (*p* = 0.016); females accounted for a larger proportion of the LBP group (64.0%), whereas males predominated in the control group (70.0%). Participant characteristics are presented in Table 1.

### 3.3. Unilateral Balance Performance

**Figure 7:**
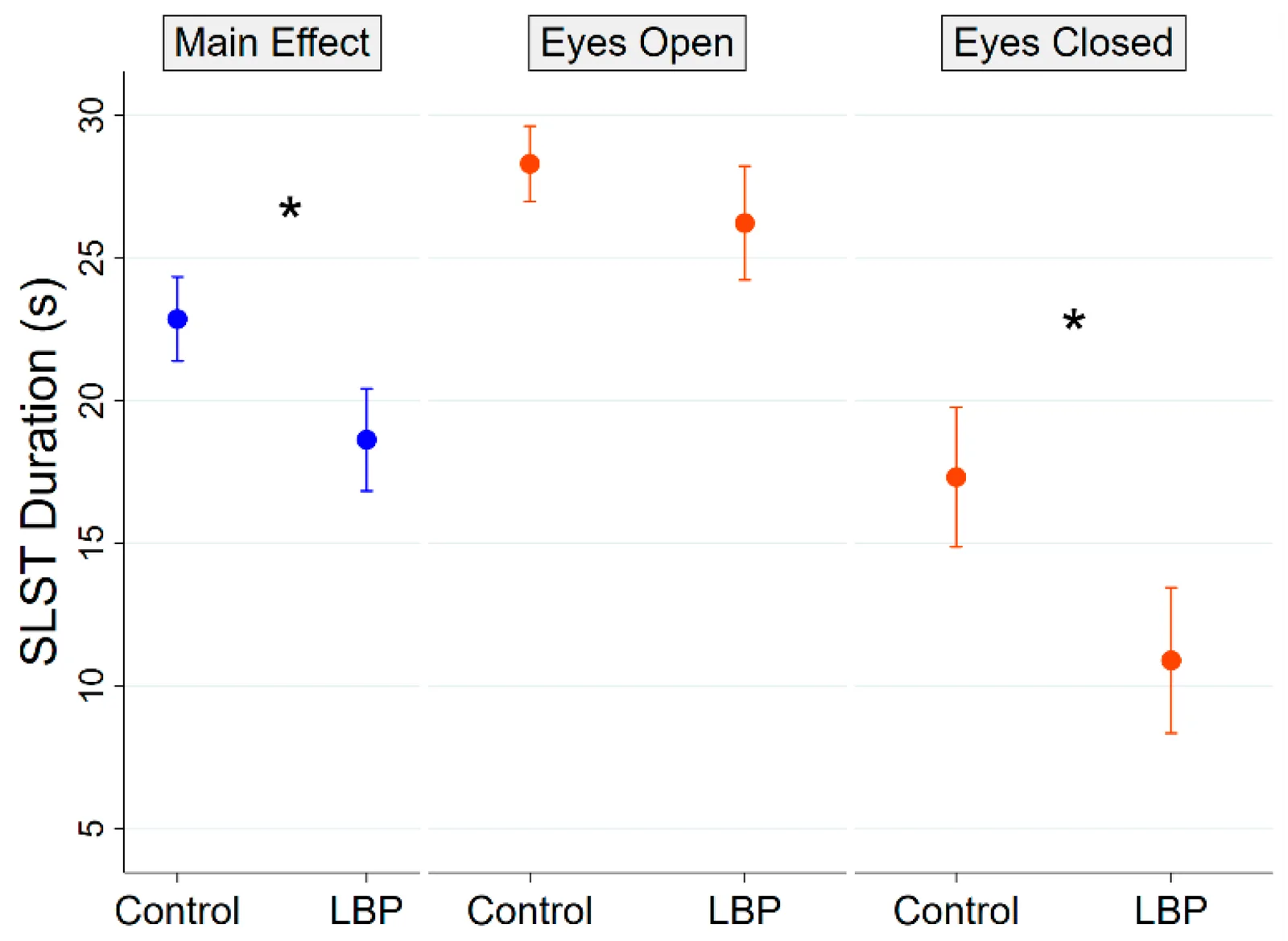
Right SLST duration estimated from the adjusted generalized estimating equations model. Results are presented for participants with and without LBP, including the overall group effect and group-specific comparisons with vision available (eyes open) and removed (eyes closed). Error bars indicate 95% confidence intervals. Note: SLST = single-leg stance test; LBP = low back pain. Asterisks (*) indicate statistically significant between-group differences.

**Figure 8:**
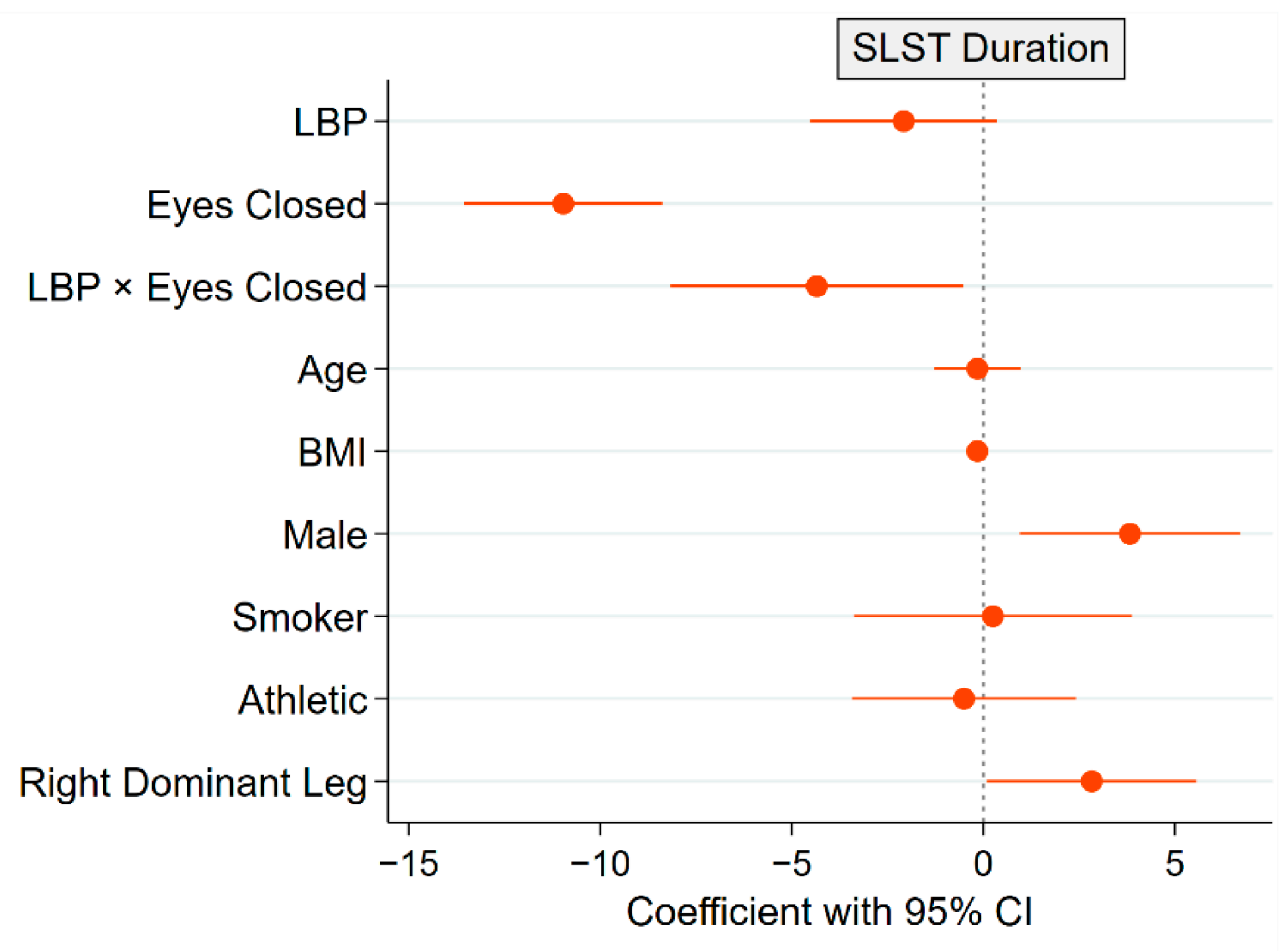
Estimated coefficients from the adjusted generalized estimating equations model for right SLST duration. Results are presented for participant group (LBP vs. control), visual condition, the group-by-visual condition interaction, and the adjustment variables. Error bars indicate 95% confidence intervals. Note: SLST = single-leg stance test; LBP = low back pain; BMI = body mass index.

For the right SLST, adjusted GEEs showed a significant main effect of group (*p* < 0.001), with participants with LBP exhibiting shorter SLST duration than participants without LBP across visual conditions (marginal mean: 18.63 s, 95% CI 16.83 to 20.42 vs. 22.86 s, 95% CI 21.38 to 24.33, respectively; Figure 7). A significant group-by-visual condition interaction was also observed (β = −4.34, 95% CI −8.16 to −0.52; *p* = 0.0258; Figure 8), indicating that the between-group difference in SLST duration was greater in the eyes-closed condition than in the eyes-open condition. Bonferroni-adjusted pairwise comparisons indicated no significant between-group difference in SLST duration under the eyes-open condition (*p* = 0.573). However, under the eyes-closed condition, participants with LBP demonstrated significantly shorter SLST duration than participants without LBP (marginal mean: 10.90 s, 95% CI 8.36 to 13.44 vs. 17.32 s, 95% CI 14.88 to 19.77, respectively; *p* = 0.002; Figure 7).

Visual condition also showed a significant main effect, with right SLST duration being shorter in the eyes-closed condition than in the eyes-open condition (marginal mean: 14.44 s, 95% CI 12.68 to 16.19 vs. 27.35 s, 95% CI 26.22 to 28.48, respectively; *p* < 0.001; Figure 8). Among the adjustment variables, being male was independently related to a longer right-sided SLST duration compared with being female (β = 3.82, 95% CI 0.95 to 6.70; *p* = 0.009; Figure 8). Right leg dominance was also associated with longer right SLST duration (β = 2.83, 95% CI 0.10 to 5.56; *p* = 0.042; Figure 8). Age, BMI, smoking status, and athletic status were not significantly associated with right-sided SLST duration in the adjusted GEE model (Figure 8).

**Figure 9:**
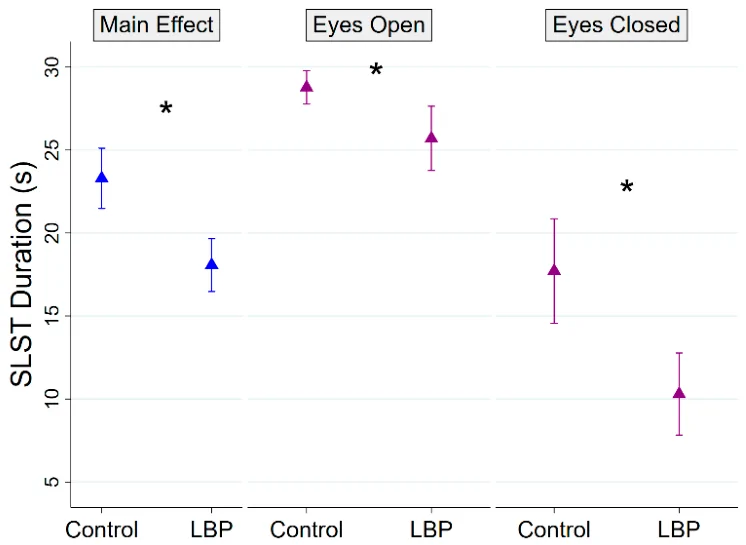
Left SLST duration estimated from the adjusted generalized estimating equations model. Results are presented for participants with and without LBP, including the overall group effect and group-specific comparisons with vision available (eyes open) and removed (eyes closed). Error bars indicate 95% confidence intervals. Note: SLST = single-leg stance test; LBP = low back pain. Asterisks (*) indicate statistically significant between-group differences.

**Figure 10:**
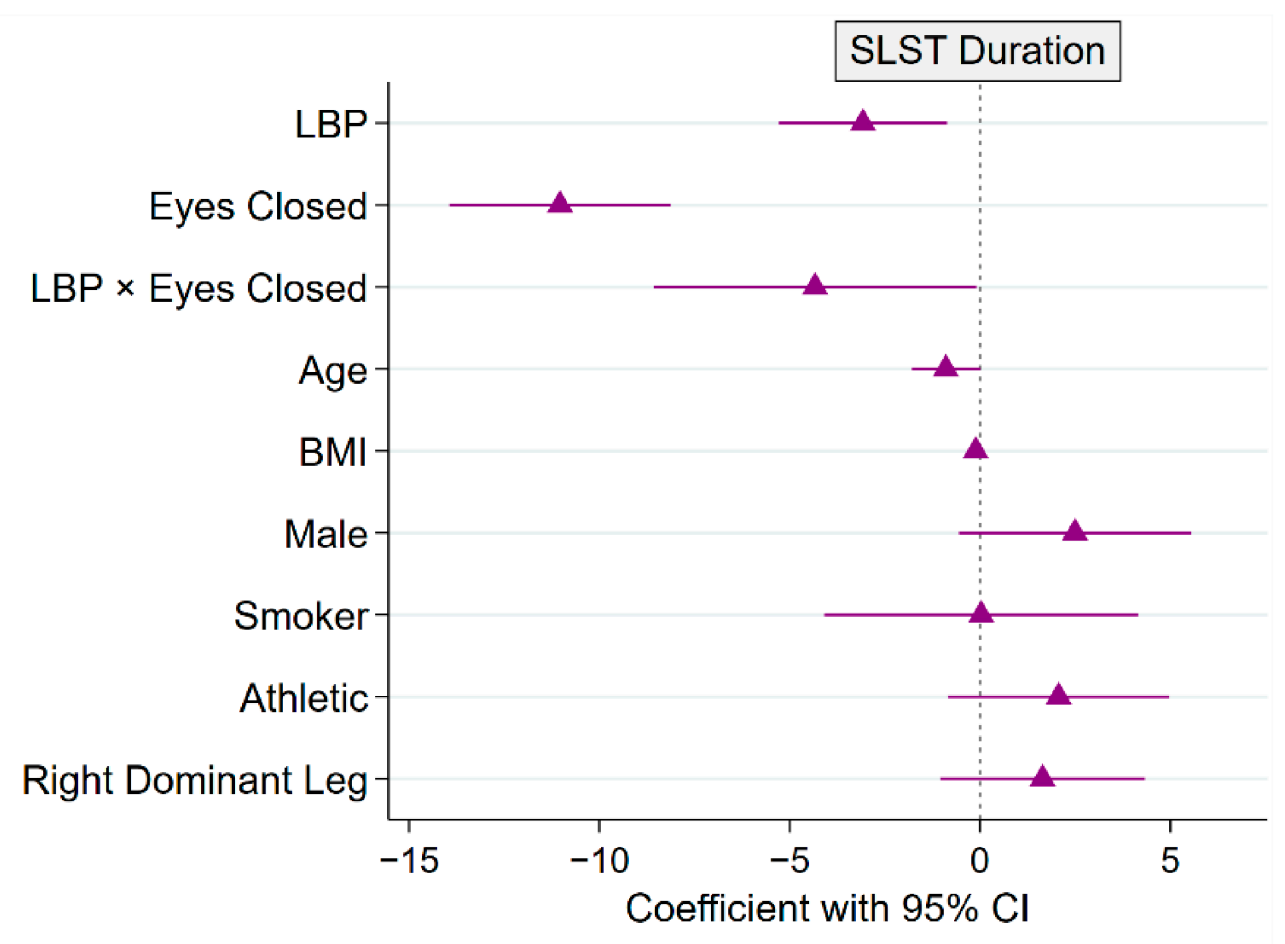
Estimated coefficients from the adjusted generalized estimating equations model for left SLST duration. Results are presented for participant group (LBP vs. control), visual condition, the group-by-visual condition interaction, and the adjustment variables. Error bars indicate 95% confidence intervals. Note: SLST = single-leg stance test; LBP = low back pain; BMI = body mass index.

For the left SLST, GEEs showed a main effect of group (*p* < 0.001), with participants with LBP exhibiting shorter SLST duration than participants without LBP across visual conditions (marginal mean: 18.07 s, 95% CI 16.48 to 19.66 vs. 23.29 s, 95% CI 21.48 to 25.11, respectively; Figure 9). A significant group-by-visual condition interaction was also observed (β = −4.33, 95% CI −8.58 to −0.09; *p* = 0.045; Figure 10), indicating that the between-group difference in SLST duration was greater in the eyes-closed condition than in the eyes-open condition. Bonferroni-adjusted pairwise comparisons indicated that participants with LBP demonstrated significantly shorter SLST duration than participants without LBP under both the eyes-open (marginal mean: 25.68 s, 95% CI 23.75 to 27.62 vs. 28.76 s, 95% CI 27.75 to 29.77; *p* = 0.038; Figure 9) and eyes-closed (marginal mean: 10.32 s, 95% CI 7.84 to 12.79 vs. 17.73 s, 95% CI 14.59 to 20.87; *p* = 0.003; Figure 9) conditions.

Visual condition also showed a significant main effect, with left SLST duration being shorter in the eyes-closed condition than in the eyes-open condition (marginal mean: 14.40 s, 95% CI 12.42 to 16.38 vs. 27.38 s, 95% CI 26.36 to 28.39, respectively; *p* < 0.001; Figure 10). Among the adjustment variables, age was independently associated with shorter left SLST duration (β = −0.90, 95% CI −1.79 to −0.004; *p* = 0.049; Figure 10). BMI, sex, smoking status, athletic status, and leg dominance were not significantly associated with left SLST duration in the adjusted GEE model (Figure 10). Complete numerical outputs from the adjusted GEE models for right and left SLST duration are provided in Supplementary Table S1.

### 3.4. Handgrip Strength

**Figure 11:**
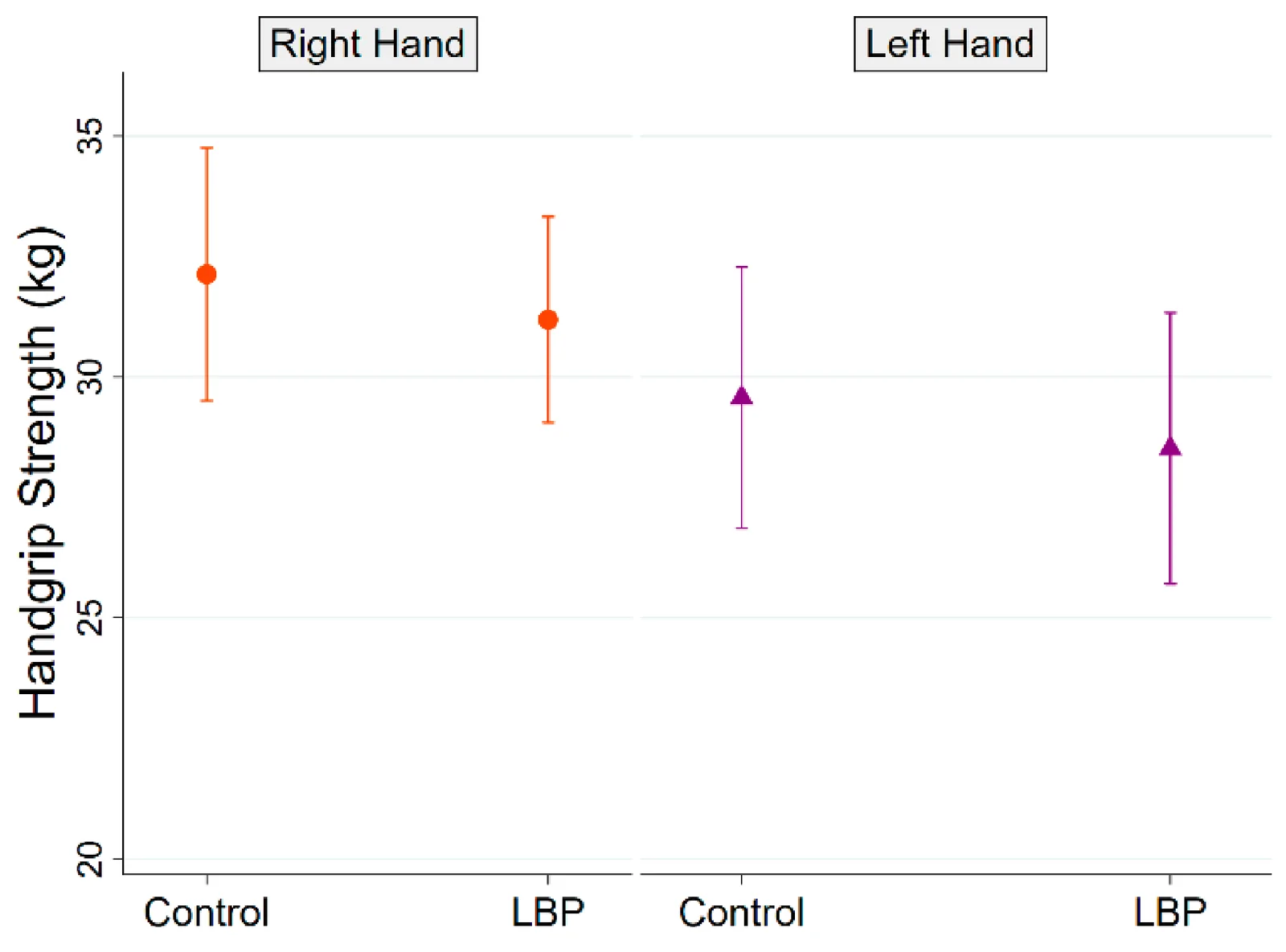
Right and left handgrip strength estimated from the adjusted multivariable linear regression models. Results are presented for participants with and without LBP. Error bars indicate 95% confidence intervals. Note: LBP = low back pain.

**Figure 12:**
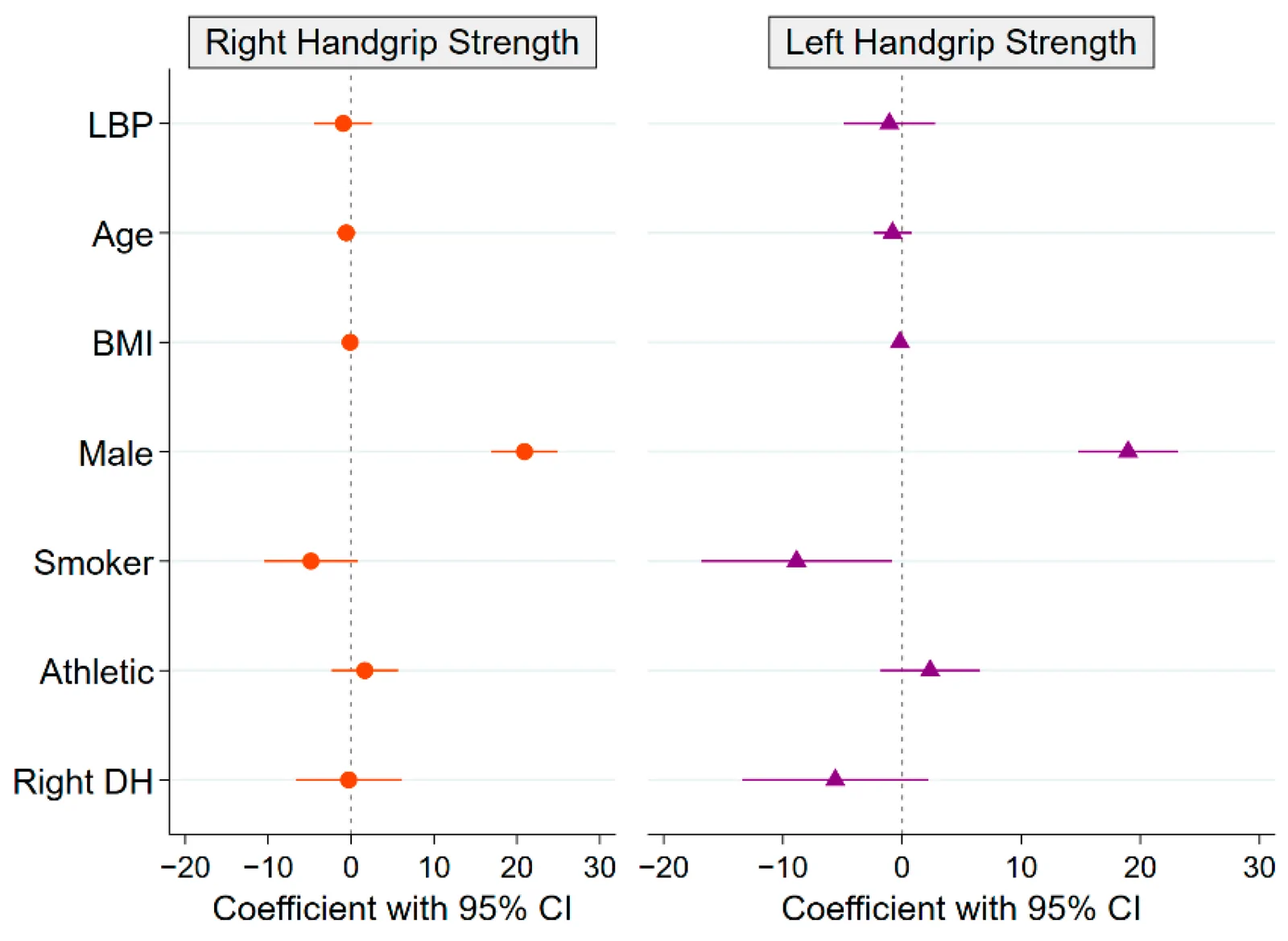
Estimated coefficients from the adjusted multivariable linear regression models for right and left handgrip strength. Results are presented for participant group (LBP vs. control) and the adjustment variables. Error bars indicate 95% confidence intervals. Note: LBP = low back pain; BMI = body mass index; DH = dominant hand.

For right handgrip strength, the unadjusted model showed a significant main effect of group (*p* = 0.010), with participants with LBP exhibiting lower handgrip strength than participants without LBP (marginal mean: 27.17 kg, 95% CI 22.64 to 31.70 vs. 35.48 kg, 95% CI 31.23 to 39.72, respectively). However, once age, BMI, sex, smoking status, athletic status, and dominant hand were accounted for in the model, the observed difference between the LBP and control groups did not reach statistical significance (*p* = 0.588). The adjusted marginal means were 31.19 kg (95% CI 29.05 to 33.32) for participants with LBP and 32.13 kg (95% CI 29.50 to 34.76) for participants without LBP (Figure 11). Among the adjustment variables, male sex was independently associated with greater right handgrip strength than female sex (β = 20.92, 95% CI 16.93 to 24.91; *p* < 0.001; Figure 12). However, no significant relationships with right handgrip strength were found for age, BMI, smoking status, athletic status, or hand dominance in the adjusted regression model (Figure 12).

For left handgrip strength, the unadjusted model also demonstrated a significant main effect of group (*p* = 0.013), with participants with LBP exhibiting lower handgrip strength than participants without LBP (marginal mean: 24.66 kg, 95% CI 19.84 to 29.49 vs. 32.78 kg, 95% CI 28.65 to 36.90, respectively). Similar to the findings for the right hand, the group effect did not remain significant following adjustment (*p* = 0.584). The adjusted marginal means were 28.51 kg (95% CI 25.70 to 31.33) for participants with LBP and 29.57 kg (95% CI 26.86 to 32.28) for participants without LBP (Figure 11). Among the adjustment variables, male sex was independently associated with greater left handgrip strength than female sex (β = 18.98, 95% CI 14.79 to 23.16; *p* < 0.001; Figure 12), whereas smoking was independently associated with lower left handgrip strength (β = −8.85, 95% CI −16.85 to −0.85; *p* = 0.031; Figure 12). However, no significant relationships with left handgrip strength were found for age, BMI, athletic status, or hand dominance in the adjusted regression model (Figure 12).

### 3.5. Foot Arch Status

**Figure 13:**
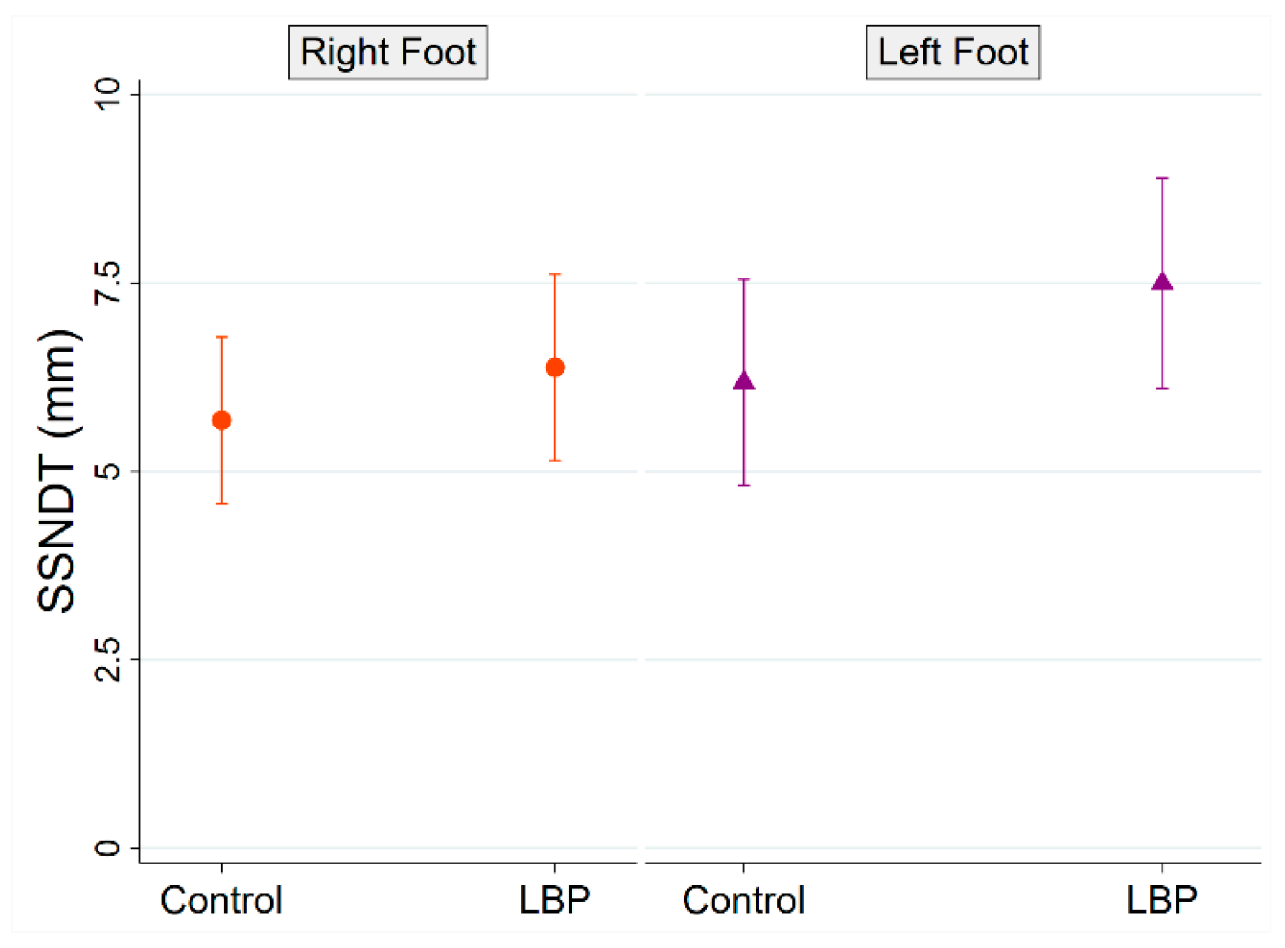
Right and left SSNDT scores estimated from the adjusted multivariable linear regression models for participants with and without LBP. Error bars indicate 95% confidence intervals. Note: SSNDT = sit-to-stand navicular drop test; LBP = low back pain.

**Figure 14:**
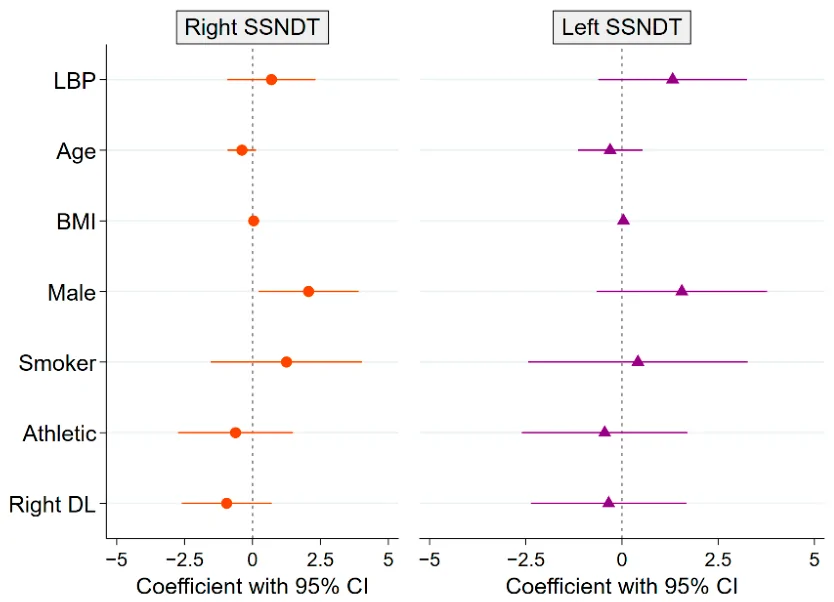
Estimated coefficients from the adjusted multivariable linear regression models for right and left SSNDT scores. Results are presented for participant group (LBP vs. control) and the adjustment variables. Error bars indicate 95% confidence intervals. Note: SSNDT = sit-to-stand navicular drop test; LBP = low back pain; BMI = body mass index; DL = dominant leg.

Both the unadjusted and adjusted models demonstrated no significant effect of group on SSNDT scores for either the right foot (unadjusted: *p* = 0.803; adjusted: *p* = 0.390) or left foot (unadjusted: *p* = 0.523; adjusted: *p* = 0.178). The adjusted marginal mean SSNDT score for the right foot was 6.38 mm (95% CI 5.15 to 7.62) in participants with LBP and 5.68 mm (95% CI 4.58 to 6.79) in participants without LBP (Figure 13). For the left foot, the adjusted marginal mean SSNDT score was 7.50 mm (95% CI 6.10 to 8.90) in participants with LBP and 6.18 mm (95% CI 4.82 to 7.55) in participants without LBP (Figure 13). Among the adjustment variables, being male was independently related to higher right-foot SSNDT scores compared with being female (β = 2.07, 95% CI 0.22 to 3.91; *p* = 0.029; Figure 14). Age, BMI, smoking status, athletic status, and dominant leg were not significantly associated with right-foot SSNDT scores (Figure 14). For the left foot, no significant relationships with SSNDT scores were found for age, BMI, sex, smoking status, athletic status, or leg dominance in the adjusted regression model (Figure 14).

### 3.6. Lower Limb Functional Capacity

**Figure 15:**
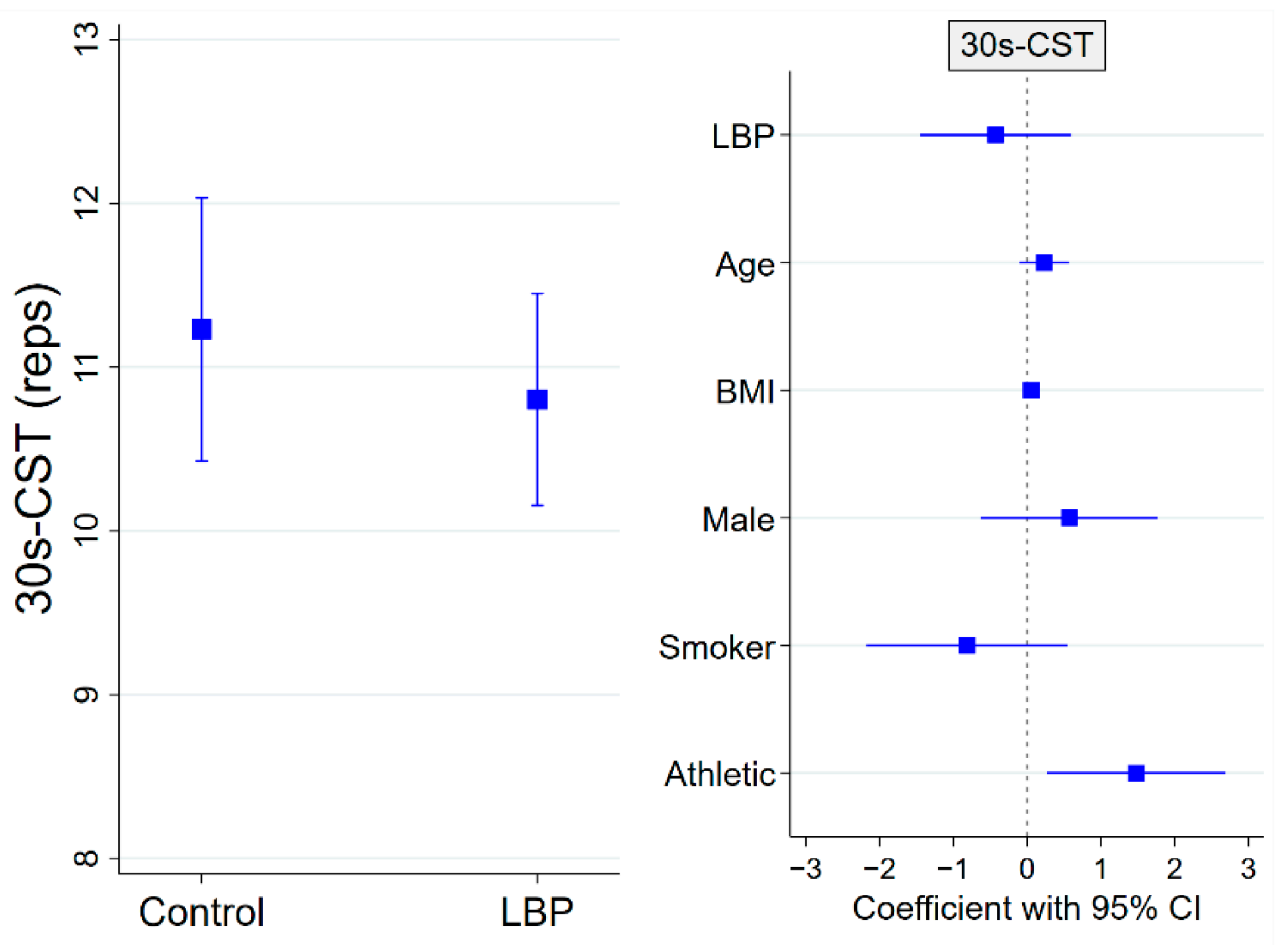
(**Left**) panel: 30s-CST performance estimated from the adjusted multivariable linear regression model for participants with and without LBP. Error bars indicate 95% confidence intervals. (**Right**) panel: estimated coefficients from the adjusted multivariable linear regression model for 30s-CST performance. Results are presented for participant group (LBP vs. control) and the adjustment variables. Error bars indicate 95% confidence intervals. Note: 30s-CST = 30-s chair stand test; LBP = low back pain; BMI = body mass index.

Both the unadjusted (*p* = 0.116) and adjusted (*p* = 0.404) models demonstrated no significant effect of group on the 30s-CST. The adjusted marginal mean number of repetitions completed within 30 s was 10.80 (95% CI 10.16 to 11.45) in participants with LBP and 11.23 (95% CI 10.43 to 12.03) in participants without LBP (Figure 15). Among the adjustment variables, athletic status independently predicted better 30s-CST performance, reflected by more repetitions achieved during the test (β = 1.48, 95% CI 0.27 to 2.69; *p* = 0.017; Figure 15). None of age, BMI, sex, or smoking status showed a significant relationship with 30s-CST performance in the adjusted regression model (Figure 15).

### 3.7. Walking Performance

**Figure 16:**
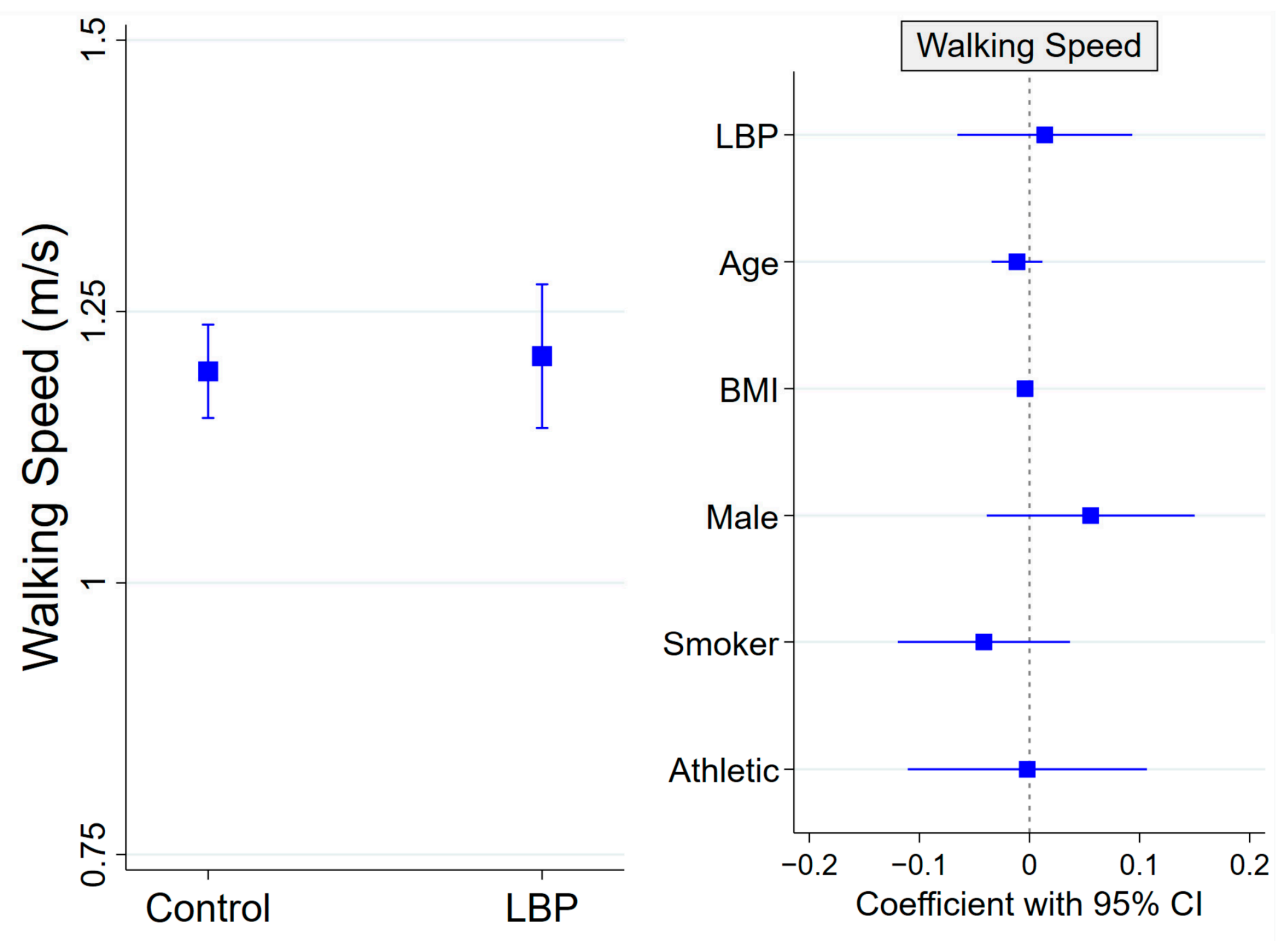
(**Left**) panel: walking speed estimated from the adjusted multivariable linear regression model for participants with and without LBP. Error bars indicate 95% confidence intervals. (**Right**) panel: estimated coefficients from the adjusted multivariable linear regression model for walking speed. Results are presented for participant group (LBP vs. control) and the adjustment variables. Error bars indicate 95% confidence intervals. Note: LBP = low back pain; BMI = body mass index.

Both the unadjusted (*p* = 0.983) and adjusted (*p* = 0.726) models demonstrated no significant effect of group on the 10-MWT. The adjusted marginal mean walking speed was 1.21 m/s (95% CI 1.14 to 1.28) in participants with LBP and 1.19 m/s (95% CI 1.15 to 1.24) in participants without LBP (Figure 16). None of age, BMI, sex, smoking status, or athletic status showed a significant relationship with walking speed in the adjusted regression model (Figure 16). Complete numerical outputs from all adjusted multivariable linear regression models are provided in Supplementary Table S2.

### 3.8. Sensitivity Analysis

All adjusted analyses were repeated with the three participants aged >35 years included. The findings were substantively unchanged. Significant group effects and group-by-visual-condition interactions remained for both right and left SLST, whereas no significant between-group differences were observed for handgrip strength, SSNDT, 10-MWT walking speed, or 30s-CST performance.

## 4. Discussion

### 4.1. Main Findings

The present study compared unilateral balance, muscular strength, arch of the foot, walking performance, and functional capacity of the lower limbs between young adults with and without LBP using multivariable analyses that accounted for important demographic, anthropometric, and lifestyle-related confounders. The principal finding was that participants with LBP demonstrated significantly poorer unilateral balance than healthy controls, with greater between-group differences under eyes-closed conditions, indicating that balance impairments were more pronounced when visual input was unavailable. In contrast, no significant differences were found between the groups for handgrip strength, arch of the foot, habitual walking speed, or functional capacity of the lower limbs after adjustment. Independent of LBP status, eyes-closed testing was consistently associated with poorer unilateral balance, while male sex was associated with better right-sided balance, greater handgrip strength, and greater right-foot navicular drop. In addition, right leg dominance was associated with better right-sided balance, increasing age with poorer left-sided balance, smoking with lower left handgrip strength, and athletic participation with better lower limb functional capacity. Overall, these findings indicate that, after accounting for important confounding factors, unilateral balance was the only functional domain that differed between young individuals with and without LBP, whereas demographic, anthropometric, and lifestyle-related characteristics influenced several measures of balance, muscular strength, foot arch status, and physical performance.

### 4.2. LBP Versus Healthy Controls

#### 4.2.1. Unilateral Balance Performance

The present study demonstrated that young adults with LBP exhibited significantly poorer unilateral balance performance than healthy controls following adjustment for relevant confounders. Importantly, the significant interaction between group and visual condition indicated that these between-group differences became more pronounced when visual input was removed, suggesting that balance impairments associated with LBP are amplified under greater sensorimotor challenge. In contrast, balance performance during the eyes-open condition was comparatively less affected. Collectively, these findings indicate that poorer unilateral balance was evident in young adults with LBP and became more apparent when postural control relied predominantly on non-visual sensory information. However, an established threshold for minimal detectable change or clinical relevance of SLST duration in comparable young adults with LBP was not identified, which limits interpretation of the observed differences in terms of their clinical magnitude.

The available evidence consistently demonstrates impaired unilateral postural control in participants with LBP compared with healthy controls using centre of pressure (CoP) variables derived from force platforms rather than SLST duration. Poorer one-leg stance performance has been reported in younger and older adults with chronic LBP, with larger impairments observed in younger individuals, particularly for CoP velocity [63]. Comparable deficits have also been reported in military special forces operators with a history of LBP [64] and competitive athletes with chronic LBP [65], who exhibited greater ground reaction force variability, CoP area, sway velocity, and total CoP displacement during one-leg stance than their counterparts without LBP. Furthermore, unilateral standing was shown to be more sensitive than bilateral standing tasks for detecting postural control impairments [66], including greater CoP area and CoP velocity, in individuals with chronic LBP. Collectively, these findings demonstrate consistent unilateral balance deficits across diverse LBP populations and support the present findings obtained using a simple clinical balance assessment.

Evidence from performance-based balance assessments further supports the present findings. Individuals with chronic LBP have demonstrated shorter single-leg stance holding time together with reduced trunk, thoracic, and lumbar standstill time, indicating impaired ability to establish and maintain stable unilateral posture [67]. Greater frequencies of early contralateral foot contacts during the initial phase of single-leg stance have also been reported with and without visual input, suggesting impaired stabilisation immediately following weight transfer [39]. Together, these findings indicate that both instrumented and performance-based assessments consistently identify deficits in unilateral postural stability among individuals with LBP.

Several mechanisms may explain the poorer unilateral balance observed in participants with LBP. Maintaining stable single-leg stance requires the combined processing of visual, vestibular, and somatosensory inputs [13] together with coordinated activation of the foot, ankle, hip, and trunk musculature to control the body’s centre of mass within a substantially decreased base of support [14,15,16]. Compared with bilateral standing, unilateral stance imposes greater demands on postural control because stability must be maintained within a smaller stability boundary while continuously responding to postural perturbations [12]. Consequently, subtle sensorimotor impairments may become more apparent during single-leg stance. Experimental evidence indicates that single-leg stance comprises two distinct phases: an initial dynamic phase, during which body weight is transferred to the stance limb and postural stability is established, followed by a static phase characterised by maintenance of a stable unilateral posture [11]. Impairments during either phase may compromise overall balance performance. Several neuromuscular impairments observed during unipedal stance in participants with LBP may contribute to poorer balance performance, including altered lumbopelvic muscle activation (e.g., reduced activation of the lumbar multifidus and iliocostalis muscles or increased trunk muscle co-activation) [68], hip dysfunction (e.g., reduced gluteus medius strength) [69], and greater susceptibility to fatigue of the lumbar extensors and hip abductors [70]. Collectively, these findings suggest that the poorer unilateral balance detected in the current study might be attributable to impaired stabilisation during the transition to single-leg support together with less efficient neuromuscular control of the lumbopelvic–hip complex during maintenance of unilateral stance. These deficits may become more apparent when visual input is removed, thereby requiring greater reliance on proprioceptive and vestibular information.

#### 4.2.2. Handgrip Strength

In contrast to unilateral balance, the between-group differences in handgrip strength were no longer statistically significant after adjustment. Although participants with LBP demonstrated lower handgrip strength in the unadjusted models, this difference was no longer evident after accounting for potential confounding variables, suggesting that it was more likely attributable to differences in participant characteristics than to LBP itself. Therefore, unlike unilateral balance, overall muscular strength, as estimated by handgrip strength, does not appear to be independently impaired in young adults with LBP. These findings further suggest that sensorimotor deficits may represent a more prominent functional characteristic of LBP than generalised reductions in muscular strength in this relatively young population.

Previous studies examining handgrip strength and LBP have reported findings broadly consistent with the present study. A population-based study of middle-aged men found that higher handgrip strength was associated with lower odds of arthralgia and back and joint stiffness, but not with LBP, suggesting that handgrip strength may not independently reflect LBP [71]. Similarly, no significant between-group differences in handgrip strength were observed among rice farmers with LBP despite lower back extensor endurance [72] and among older adults despite poorer abdominal and back muscle strength [73], suggesting that LBP-related muscular impairments may be more specific to the trunk than reflected by handgrip strength. Likewise, a population-based study of community-dwelling women aged over 60 years found no significant difference in handgrip strength according to LBP status, whereas physically inactive participants had lower handgrip strength than physically active participants, suggesting that physical activity may contribute more to grip strength than LBP status alone [74]. These findings support the interpretation that handgrip strength, although commonly utilised as an indirect indicator of general muscle strength and physical function [18,19,20,21,22,23], may provide limited insight into the trunk-specific neuromuscular impairments that characterise LBP, particularly in relatively young adults with preserved overall physical function.

#### 4.2.3. Foot Arch Status

Unlike unilateral balance, arch of the foot did not differ significantly between young adults with LBP and healthy controls after adjustment. Although altered foot posture has been proposed as a contributor to LBP, the present findings do not support a significant difference in MLA behaviour, assessed using the SSNDT, between young adults with and without LBP. Participants with LBP demonstrated slightly greater navicular drop values; however, MLA mobility in both groups fell within the generally accepted clinical reference range of approximately 5–9 mm [26,52,75], indicating broadly comparable functional foot arch behaviour.

The current results generally align with earlier research based on objective assessments of foot posture. Findings from the Framingham Foot Study showed no independent association between foot posture, assessed using the arch index, and LBP after adjustment for potential confounders [31]. Similarly, no meaningful differences in navicular drop or calcaneal eversion were identified between middle-aged individuals with recurrent mechanical LBP and healthy controls [76]. Likewise, no significant between-group differences in navicular drop were reported between individuals with LBP and healthy controls [77], despite significant differences in Foot Posture Index-6 scores, suggesting that the navicular drop test and Foot Posture Index assess different aspects of foot posture.

Several factors may explain why foot arch status did not differ significantly between the groups. First, the relatively normal MLA mobility observed in both groups suggests that mild variations in arch mobility may be insufficient to distinguish individuals with and without LBP. Consistent with this, moderate and severe pes planus, but not mild flexible pes planus, were associated with approximately twice the prevalence of intermittent LBP among military recruits [78], indicating that deformity severity may be more important than the presence of a flattened arch alone. Second, navicular drop quantifies MLA mobility but may not capture the dynamic biomechanical adaptations associated with LBP. Consistent with this, the Framingham Foot Study found no independent association between static foot posture and LBP, whereas dynamic foot function during walking was independently associated with LBP in women [31]. Likewise, individuals with excessive foot pronation exhibited comparable navicular drop measurements regardless of LBP status, yet those with LBP demonstrated altered ground reaction forces, lower limb biomechanics, and muscle activation during walking [32,33]. Collectively, these findings suggest that dynamic foot function and neuromuscular control may better reflect foot-related biomechanical alterations associated with LBP than navicular drop alone, particularly in relatively young adults with MLA mobility within normal clinical limits.

#### 4.2.4. Lower Limb Functional Capacity

Functional capacity of the lower limbs, assessed using the 30s-CST, did not differ significantly between young adults with LBP and healthy controls after adjustment for potential confounding variables. These findings indicate that lower limb functional capacity remained largely preserved in young adults with LBP. The present results are broadly consistent with earlier studies using the 30s-CST or similar functional chair-rise assessments. No significant between-group differences in 30s-CST performance were reported among community-dwelling older adults despite poorer abdominal and back muscle strength in women with LBP [73]. Likewise, a population-based study of older women found that LBP status was not independently associated with chair-rise performance, whereas physical inactivity and greater pain intensity were more important determinants of performance [74]. In contrast, studies using timed five-repetition sit-to-stand protocols have consistently reported poorer performance in individuals with LBP [79,80,81]. Unlike the 30s-CST, where performance is determined by how many sit-to-stand repetitions are achieved over 30 s, the five-repetition sit-to-stand test evaluates the duration needed to perform five successive repetitions. Consequently, the two tests assess different aspects of lower limb function, with the latter emphasising movement speed under maximal effort and the former reflecting functional muscular endurance.

The preserved 30s-CST performance observed in the present study suggests that repeated sit-to-stand ability may remain relatively unaffected in young adults with LBP. This is consistent with evidence indicating that impaired 30s-CST performance has been reported predominantly in older adults or individuals with disability, in whom poorer performance is more closely related to reduced functional mobility and disability than to LBP itself [82]. Nevertheless, preserved task performance does not necessarily indicate normal movement strategies. Previous kinematic research has demonstrated that individuals with chronic LBP exhibit reduced lumbar lordosis throughout sit-to-stand and stand-to-sit movements compared with healthy controls despite similar initial sitting posture, suggesting altered movement patterns during task execution [83]. Furthermore, psychological factors such as kinesiophobia have been independently associated with poorer 30s-CST performance in older adults with chronic LBP [84], although these factors were not assessed in the current study. Collectively, these findings suggest that preserved 30s-CST performance does not necessarily exclude underlying biomechanical or psychological adaptations associated with LBP.

#### 4.2.5. Walking Performance

Walking performance did not differ significantly between young adults with LBP and healthy controls after adjustment for potential confounding variables. These results imply that habitual walking speed remained largely preserved in young individuals with LBP. The current results are broadly consistent with evidence indicating that the presence of LBP alone may not be sufficient to impair habitual walking speed. Population-based studies have reported no independent association between LBP status and habitual gait speed when relevant confounding factors were accounted for, whereas higher pain intensity remained independently related to poorer walking performance [74,79]. Similarly, several laboratory- and treadmill-based studies have reported no significant between-group differences in self-selected walking speed or most spatiotemporal gait characteristics between individuals with mild-to-moderate LBP and healthy controls [85,86,87]. In contrast, slower walking performance has generally been observed in individuals with chronic or more disabling LBP, particularly in the presence of referred leg pain, greater disability, or higher pain intensity [85,88,89]. These findings also suggest that walking performance varies according to clinical characteristics within the LBP population rather than the presence of LBP alone. Individuals classified as low risk of persistent disability using the STarT Back Tool demonstrated faster walking speeds and better physical performance than those classified as medium or high risk [90]. Similarly, psychological factors, including fear-avoidance beliefs, have been associated with slower walking speed and greater gait variability in participants with chronic LBP [91,92]. Collectively, these findings suggest that habitual walking speed is influenced more by symptom severity, psychosocial factors, and clinical presentation than by the presence of LBP alone, which may explain the preserved walking performance observed in the present relatively young cohort.

### 4.3. Effects of Categorical Factors and Continuous Covariates

The marked decline in SLST duration when vision was removed demonstrates the important role of visual information in preserving single-leg balance. Across both adjusted GEE models, removal of visual feedback resulted in substantially shorter SLST duration, indicating that successful performance during single-leg stance depends heavily on visual information. These findings align with earlier research reporting substantial reductions in SLST performance when vision is removed because postural control becomes increasingly dependent on somatosensory, vestibular, and neuromuscular inputs [10,40,93]. They are also consistent with evidence indicating that individuals with musculoskeletal disorders (e.g., ankle and knee injuries) depend more heavily on visual information to offset deficits in proprioceptive and sensorimotor function, thereby making balance deficits more apparent under eyes-closed conditions [17,94]. Collectively, these findings reinforce the importance of evaluating unilateral balance under both visual conditions, as assessment under eyes-open conditions alone may underestimate subtle deficits in postural control.

Sex-related differences were also evident across several outcome measures. Male participants demonstrated longer SLST duration, greater handgrip strength, and greater SSNDT scores than female participants. The observed sex differences in handgrip strength (both hands) and, to a lesser extent, unilateral balance (right foot) are biologically plausible and are consistent with evidence demonstrating greater muscle strength in males. These differences are primarily attributed to greater skeletal muscle mass, larger muscle fibre cross-sectional area, and greater absolute force-generating capacity [95,96]. In addition, men generally participate in strength training more frequently and preferentially engage in higher-intensity and competitive exercise [95], which may further enhance muscular performance and functional capacity. Beyond the observed differences in muscular performance and unilateral balance, males also demonstrated greater SSNDT scores (right foot) than females. This finding is consistent with previous evidence [97,98] demonstrating greater navicular drop in males, which has been attributed to sex-related differences in foot dimensions, foot morphology, and the biomechanics of the MLA during weight bearing. Age was associated with shorter SLST duration (left foot). However, this finding warrants caution because the study population covered a limited age range (19–25 years).

Because previous evidence indicates that meaningful age-related deterioration in single-leg balance generally occurs much later in adulthood [99], the present finding is unlikely to reflect physiological aging and may instead represent normal individual variability within this young adult cohort. Athletic status was also associated with lower limb functional capacity, with athletic participants completing significantly more repetitions during the 30s-CST than non-athletic participants. A previous Saudi study likewise reported superior walking speed and agility among female basketball players compared with non-athletic controls [100]. This association is biologically plausible because regular participation in sport and structured physical training is associated with greater lower limb muscle strength, enhanced proprioceptive acuity, improved neuromuscular control, and more accurate force generation [101,102,103], all of which contribute to repeated sit-to-stand performance.

Smoking was associated with lower left handgrip strength. Although this finding should be interpreted cautiously because relatively few participants were smokers and the association was not evident for the right hand, it is consistent with previous evidence [59] demonstrating that tobacco smoking adversely affects skeletal muscle health and is associated with reduced muscle strength. Right leg dominance was also associated with longer right SLST duration. This finding is biologically plausible because the dominant limb is used more frequently during functional activities, which may promote superior neuromuscular coordination and postural control [60]. The high proportion of participants with right leg dominance in the study sample may further explain why this association was evident only for the right limb, as the dominant limb was predominantly the right limb in the present population. Recent computational evidence further demonstrates that lower-limb asymmetry can produce limb-specific alterations in mechanical loading, joint kinetics, and muscle activation during locomotion [104]. Although these findings were derived from running and cannot be directly extrapolated to static unilateral balance, they provide complementary biomechanical context suggesting that functional differences between limbs may be accompanied by distinct neuromuscular and mechanical demands.

Overall, these secondary findings indicate that participant characteristics influenced specific functional outcomes independently of LBP status. However, these associations were generally outcome- and limb-specific rather than consistently observed across all assessments, suggesting that unilateral balance, muscular strength, foot arch behaviour, and lower limb functional capacity are influenced by distinct physiological and biomechanical mechanisms rather than a common underlying factor. Associations involving individual adjustment covariates should be considered exploratory and interpreted cautiously in view of the number of model terms examined.

### 4.4. Clinical Implications

The findings of this study may have several implications for clinical practice. First, unilateral balance assessment, particularly under eyes-closed conditions, may provide additional clinically relevant information when evaluating young adults with LBP, as unilateral balance was the only functional domain that differed between groups after adjustment for important demographic, anthropometric, and lifestyle-related factors. Accordingly, the use of the SLST with visual input available and removed may complement routine clinical assessment by identifying balance impairments associated with LBP in this population. In contrast, although no between-group differences were observed for handgrip strength, arch of the foot, habitual walking speed, or functional capacity of the lower limbs, these findings should not be interpreted as indicating that these measures lack clinical value. Rather, within this cohort of young adults, no statistically significant between-group differences were detected in these outcomes after accounting for potential confounding factors. Finally, the associations observed for sex, age, smoking status, athletic participation, and limb dominance further emphasise the importance of considering individual participant characteristics when interpreting sensorimotor and functional assessment findings in young adults with LBP.

### 4.5. Limitations

Several limitations need to be taken into account when interpreting the results. First, the cross-sectional nature of the study does not allow the direction of the association or causality between LBP and the identified balance deficits to be established. Second, the study was restricted to young Saudi adults, and the findings may therefore not be directly generalisable to older adults or to individuals with LBP presenting across a broader spectrum of clinical severity. Third, although the analyses accounted for several important demographic, anthropometric, and lifestyle-related confounding factors, detailed LBP-specific characteristics, such as pain-related disability and psychological factors, were not evaluated and may also influence functional performance. Fourth, postural balance was assessed using established clinical measures rather than laboratory-based biomechanical assessments. Although the SLST is a valid and widely used clinical assessment with demonstrated reliability, instrumented measures such as force platforms or wearable motion sensors may provide additional insight into the biomechanical mechanisms underlying the observed balance impairments. Fifth, the upper age restriction (>35 years) was applied after enrolment to maintain a relatively homogeneous young-adult sample, as all other participants were aged 18–25 years. However, sensitivity analyses including these three participants yielded substantively unchanged findings. Sixth, information on LBP duration, recurrence, previous episodes, referred symptoms, and disability was not collected; therefore, participants could not be classified according to acute, subacute, chronic, or recurrent LBP, which may have introduced clinical heterogeneity within the LBP group. Seventh, limiting the SLST to 30 s may have resulted in a ceiling effect, especially during eyes-open testing, and should be considered when interpreting these findings. These limitations should be considered alongside the strengths of the study, including standardized testing procedures, established outcome measures, assessment of single-leg balance with visual input available and removed, adjustment for multiple potential confounders, and adequately powered multivariable analyses.

## 5. Conclusions

In conclusion, young adults with LBP demonstrated poorer unilateral balance than healthy controls, with greater between-group differences observed under eyes-closed conditions, whereas handgrip strength, arch of the foot, walking speed, and functional capacity of the lower limbs did not differ between groups. These findings provide novel evidence from Saudi Arabia and suggest that assessment of unipedal balance with visual input available and removed may provide potentially relevant information when evaluating young adults with LBP. However, its clinical utility requires confirmation in prospective studies. Future longitudinal studies involving larger samples and individuals across different age groups are warranted to confirm these findings and determine whether unilateral balance impairments predict clinically important outcomes. In addition, future studies should incorporate comprehensive biopsychosocial assessments together with objective biomechanical and instrumented measures of postural control to better characterise the mechanisms underlying impaired unilateral balance and determine its clinical significance across the spectrum of LBP.

## Figures and Tables

**Table 1:** Characteristics of participants with and without LBP.

Characteristics		Mean ± SD or Median (IQR)—N (%)			*p* Value
		Both (N = 55)	LBP (N = 25)	Control (N = 30)	
Age		21 (20–22)	21 (20–22)	21 (20–21)	0.5444
Height (cm)		165.7 ± 8.8	164.3 ± 8.4	166.9 ± 9.2	0.2755
Weight (kg)		65.5 (52.9–82.5)	59.1 (49.7–74.5)	70.3 (58.2–88.5)	0.0926
BMI (kg/m^2^)		23.6 (20.0–29.3)	23.5 (19.3–27.7)	24.3 (21.3–30.9)	0.2610
NPRS		-	5.1 ± 1.5	-	-
Sex	Male	30 (54.55)	9 (36.00)	21 (70.00)	**0.016**
	Female	25 (45.45)	16 (64.00)	9 (30.00)	
Smoking Status	Smoker	8 (14.55)	4 (16.00)	4 (13.33)	1.000
	Non-smoker	47 (85.45)	21 (84.00)	26 (86.67)	
Athletic status	Yes	21 (38.18)	8 (32.00)	13 (43.33)	0.419
	No	34 (61.82)	17 (68.00)	17 (56.67)	
Dominant leg	Right	39 (70.91)	19 (76.00)	20 (66.67)	0.556
	Left	16 (29.09)	6 (24.00)	10 (33.33)	
Dominant hand	Right	49 (89.09)	23 (92.00)	26 (86.67)	0.678
	Left	6 (10.91)	2 (8.00)	4 (13.33)	

Abbreviations: LBP, low back pain; SD, standard deviation; IQR, interquartile range; N, number of participants; %, percent; cm, centimetres; kg, kilograms; BMI, body mass index; kg/m^2^, kilograms per square metre; NPRS, numeric pain rating scale. Statistics: Group differences in continuous variables were examined using independent two-sample *t*-tests, while categorical variables were analysed with Fisher’s exact test. For continuous variables that did not meet normality assumptions (age, weight, and BMI), the Wilcoxon rank-sum test was used. Values reaching statistical significance (*p* < 0.05) are shown in bold.

## Data Availability

The original contributions presented in this study are included in the article; further inquiries can be directed to the corresponding author.

## Supplementary Materials

The following supporting information can be downloaded at: https://www.mdpi.com/article/10.3390/jcm15176648/s1. Table S1: Numerical outputs from the adjusted GEE models for right and left SLST; Table S2: Numerical outputs from the adjusted multivariable linear regression models for handgrip strength, SSNDT, 10-MWT, and 30s-CST.

## Abbreviations

The following abbreviations are used in this manuscript:

LBP	Low back pain
BMI	Body mass index
SLST	Single-Leg Stance Test
SSNDT	Sit-to-Stand Navicular Drop Test
MLA	Medial longitudinal arch
10-MWT	10-Metre Walk Test
30s-CST	30-Second Chair Stand Test
STROBE	Strengthening the Reporting of Observational Studies in Epidemiology
NPRS	Numeric Pain Rating Scale
kg/m^2^	Kilograms per square metre
ICC	Intraclass correlation coefficient
m/s	Metres per second
SD	Standard deviation
IQR	Interquartile range
GEEs	Generalized estimating equations
CIs	Confidence intervals
N	Number of participants
%	Percent
cm	Centimetres
kg	Kilograms
DH	Dominant hand
DL	Dominant leg
CoP	Centre of pressure
