# Supplementary material for: Unilateral Balance, Muscular Strength, Foot Arch Status, and Physical Performance in Young Adults with and Without Low Back Pain
Source: J Clin Med. 2026 Aug 28;15(17):6648. doi: 10.3390/jcm15176648 (PMC13566785; doi:10.3390/jcm15176648)
Supplement: Supplementary file 1 [file jcm-15-06648-s001.zip › jcm-4490557-supplementary.pdf]

Table S1. Numerical outputs from the adjusted GEE models for right and left SLST.

Outcome	Model Terms	Coefficient (95% CI)	P value	Model Statistic
Right SLST	LBP group	−2.08 (−4.52 to 0.36)	0.095	Wald χ^2 = 326.67
	Eyes closed	−10.96 (−13.54 to −8.38)	<0.001	
	LBP × eyes closed	−4.34 (−8.16 to −0.52)	**0.026**	
	Age	−0.15 (−1.28 to 0.97)	0.788	
	BMI	−0.16 (−0.34 to 0.02)	0.088	
	Male sex	3.82 (0.95 to 6.70)	**0.009**	
	Smoker	0.25 (−3.38 to 3.88)	0.892	
	Athlete	−0.50 (−3.43 to 2.42)	0.735	
	Right leg dominance	2.83 (0.10 to 5.56)	**0.042**	
Left SLST	LBP group	−3.08 (−5.29 to −0.87)	**0.006**	Wald χ^2 = 312.71
	Eyes closed	−11.03 (−13.94 to −8.13)	**<0.001**	
	LBP × eyes closed	−4.33 (−8.58 to −0.09)	**0.045**	
	Age	−0.90 (−1.79 to −0.004)	**0.049**	
	BMI	−0.11 (−0.30 to 0.08)	0.251	
	Male sex	2.50 (−0.56 to 5.55)	0.109	
	Smoker	0.03 (−4.09 to 4.16)	0.988	
	Athlete	2.07 (−0.84 to 4.98)	0.164	
	Right leg dominance	1.64 (−1.04 to 4.33)	0.230	

Abbreviations: GEE, generalized estimating equation; SLST, single-leg stance test; CI, confidence interval; LBP, low back pain; BMI, body mass index.

Table S2. Numerical outputs from the adjusted multivariable linear regression models for handgrip strength, SSNDT, 10-MWT, and 30s-CST.

Outcome	Model Terms	Coefficient (95% CI)	P value	Model Statistic
Right handgrip strength	LBP group	−0.94 (−4.42 to 2.54)	0.588	$R^2 = 0.771$
	Age	−0.58 (−1.71 to 0.54)	0.302	
	BMI	−0.11 (−0.36 to 0.15)	0.407	
	Male sex	20.92 (16.93 to 24.91)	**<0.001**	
	Smoker	−4.84 (−10.48 to 0.81)	0.091	
	Athlete	1.66 (−2.36 to 5.68)	0.411	
	Right hand dominance	−0.28 (−6.68 to 6.12)	0.930	
Left handgrip strength	LBP group	−1.06 (−4.91 to 2.80)	0.584	$R^2 = 0.689$
	Age	−0.79 (−2.40 to 0.81)	0.326	
	BMI	−0.18 (−0.57 to 0.22)	0.371	
	Male sex	18.98 (14.79 to 23.16)	**<0.001**	
	Smoker	−8.85 (−16.85 to −0.85)	**0.031**	
	Athlete	2.36 (−1.82 to 6.55)	0.262	
	Right hand dominance	−5.60 (−13.42 to 2.23)	0.157	
Right SSNDT	LBP group	0.70 (−0.92 to 2.32)	0.390	$R^2 = 0.186$
	Age	−0.39 (−0.91 to 0.13)	0.141	
	BMI	0.04 (−0.09 to 0.18)	0.528	
	Male sex	2.07 (0.22 to 3.91)	**0.029**	
	Smoker	1.25 (−1.54 to 4.04)	0.372	
	Athlete	−0.62 (−2.74 to 1.49)	0.557	
	Right-leg dominance	−0.95 (−2.60 to 0.71)	0.256	
Left SSNDT	LBP group	1.32 (−0.62 to 3.25)	0.178	$R^2 = 0.080$
	Age	−0.31 (−1.15 to 0.53)	0.467	
	BMI	0.04 (−0.08 to 0.16)	0.513	
	Male sex	1.55 (−0.67 to 3.77)	0.166	
	Smoker	0.41 (−2.44 to 3.27)	0.772	
	Athlete	−0.45 (−2.60 to 1.70)	0.677	
	Right-leg dominance	−0.34 (−2.37 to 1.68)	0.733	
10-MWT	LBP group	0.014 (−0.065 to 0.093)	0.726	$R^2 = 0.076$
	Age	−0.011 (−0.034 to 0.012)	0.328	
	BMI	−0.004 (−0.010 to 0.002)	0.215	
	Male sex	0.056 (−0.039 to 0.150)	0.242	
	Smoker	−0.041 (−0.120 to 0.037)	0.293	
	Athlete	−0.002 (−0.111 to 0.107)	0.971	
30s-CST	LBP group	−0.43 (−1.45 to 0.59)	0.404	$R^2 = 0.220$
	Age	0.23 (−0.11 to 0.57)	0.175	
	BMI	0.05 (−0.05 to 0.16)	0.307	
	Male sex	0.57 (−0.63 to 1.77)	0.342	
	Smoker	−0.81 (−2.18 to 0.55)	0.236	
	Athlete	1.48 (0.27 to 2.69)	**0.017**	

Abbreviations: SSNDT, sit-to-stand navicular drop test; 10-MWT, 10-meter walk test; 30s-CST, 30-second chair stand test, confidence interval; LBP, low back pain; BMI, body mass index.
